# Involvement of Arabidopsis Multi-Copper Oxidase-Encoding *LACCASE12* in Root-to-Shoot Iron Partitioning: A Novel Example of Copper-Iron Crosstalk

**DOI:** 10.3389/fpls.2021.688318

**Published:** 2021-10-11

**Authors:** María Bernal, Ute Krämer

**Affiliations:** ^1^Department of Molecular Genetics and Physiology of Plants, Faculty of Biology and Biotechnology, Ruhr University Bochum, Bochum, Germany; ^2^Department of Plant Nutrition, Estación Experimental de Aula Dei-CSIC, Zaragoza, Spain

**Keywords:** copper, iron, multicopper oxidase, homeostasis, deficiency

## Abstract

Numerous central biological processes depend on the participation of the essential elements iron (Fe) or copper (Cu), including photosynthesis, respiration, cell wall remodeling and oxidative stress protection. Yet, both Fe and Cu metal cations can become toxic when accumulated in excess. Because of the potent ligand-binding and redox chemistries of these metals, there is a need for the tight and combined homeostatic control of their uptake and distribution. Several known examples pinpoint an inter-dependence of Fe and Cu homeostasis in eukaryotes, mostly in green algae, yeast and mammals, but this is less well understood in multicellular plants to date. In Arabidopsis, Cu deficiency causes secondary Fe deficiency, and this is associated with reduced *in vitro* ferroxidase activity and decreased root-to-shoot Fe translocation. Here we summarize the current knowledge of the cross-talk between Cu and Fe homeostasis and present a partial characterization of *LACCASE12* (*LAC12*) that encodes a member of the multicopper oxidase (MCO) protein family in Arabidopsis. *LAC12* transcript levels increase under Fe deficiency. The phenotypic characterization of two mutants carrying T-DNA insertions suggests a role of *LAC12* in root-to-shoot Fe partitioning and in maintaining growth on Fe-deficient substrates. A molecular understanding of the complex interactions between Fe and Cu will be important for combating Fe deficiency in crops and for advancing biofortification approaches.

## Introduction

Copper (Cu) and iron (Fe) are essential micronutrients for plants and most other forms of life. Both elements function as cofactors of important enzymes in a large number of biochemical pathways due to their redox chemical properties, as for example the electron transport chains involved in photosynthesis and mitochondrial respiration (Nouet et al., 2011; Ravet and Pilon, 2013). Fe also plays an important role in chlorophyll biosynthesis, sulfate assimilation, hormone signaling, nitrogen metabolism and DNA synthesis and repair. Cu is also required in ethylene perception, cell wall metabolism and oxidative stress responses (Printz et al., 2016). The importance of both micronutrients is even more evident through the deleterious effects that their corresponding deficiencies provoke in plants. Common symptoms of Cu deficiency are plant chlorosis in young tissues and necrosis in reproductive organs (Tewari et al., 2006; Printz et al., 2016). Fe deficiency causes severe chlorosis, and is a major restriction for crop yield and nutritional quality (Marschner, 1995; Puig et al., 2007; Connorton et al., 2017; Kroh and Pilon, 2020). The same redox properties of Cu and Fe that are crucial for the correct function of a wide range of proteins, can become toxic when these metals are accumulated in excess. Both excess Fe and excess Cu can induce the uncontrolled production of reactive oxygen species (ROS), combined with between-metal interference and competition (Ravet and Pilon, 2013). Consequently, there is a need for a tight homeostatic control of uptake and distribution of both metals.

The impact of plant nutrition on human health is large. The World Health Organization (WHO) estimates that 30% of the world’s population^1^ are not taking in adequate amounts of Fe and therefore suffering from Fe-deficiency anemia. In infancy, for example, insufficient Fe intake can result in cognitive limitations that persist into adulthood (Collins et al., 2010). Additionally, it is well known that Fe and Cu also interact in the human diet. Both Fe and Cu deficiency can co-occur in selected populations consuming normal diets (Collins et al., 2010). To handle better these micronutrient deficiencies, a number of different strategies are being developed, as for instance oral additives, enriching prepared foods by adding supplements, and spraying diverse crop plants with fertilizers containing Cu or Fe (Zhao and McGrath, 2009; Shahzad et al., 2014). Another approach is to screen plants for identifying varieties with enhanced levels of Fe or Cu in their grains or in other edible tissues, followed if necessary, by using certain genetic markers to later breed only with chosen varieties. This approach is known as biofortification. Targeted biofortification approaches are also promising, as exemplified in the study in which increased production of ferritins or nicotianamine synthases could help to increase the Fe content in rice grains (Masuda et al., 2013). However, the accumulation of excess concentrations of the same essential metals can also cause human diseases as for example Wilson’s disease (Cu) or hemochromatosis (Fe) (Dusek et al., 2015). Thus, these metals are a risk for human health. Additionally, overaccumulation of these metals can contaminate plants, livestock and wildlife (Nriagu and Pacyna, 1988). The position of plants at the beginning of the food chain implies that an improved understanding of metal homeostasis of higher plants is fundamentally important for the environment, agriculture and human health.

Higher plants have established systemic homeostatic processes to efficiently take up, distribute and use micronutrients such as Cu and Fe. The molecular mechanisms of Cu and Fe homeostasis are quite well understood individually. However, an understanding of the cross-talk among the homeostasis of these metals is only beginning to arise (Table 1). Metalloprotein substitution is one well-characterized example of cross-talk between Cu and Fe homeostasis in higher plants (Yamasaki et al., 2007; Waters et al., 2012; Pilon, 2017). In *Arabidopsis thaliana*, when Cu is scarce, CU/ZN SUPEROXIDE DISMUTASES 1 and 2 (CSD1 and CSD2) are replaced by FE SUPEROXIDE DISMUTASE 1 (FSD1), most likely to reorganize metabolism to economize on Cu for maintaining the functions of essential proteins such as PLASTOCYANIN (PC). A metalloprotein substitution mechanism of economizing on Cu was first identified in the green alga *Chlamydomonas reinhardtii.* When this green alga grows under Cu deficiency conditions, plastocyanin is replaced by the haem-containing cytochrome *c*_6_ (Quinn and Merchant, 1995; Kropat et al., 2005). This substitution mechanism is regulated by the transcription factor COPPER RESPONSE REGULATOR (CRR1) in Chlamydomonas. In Arabidopsis, the homologous SQUAMOSA PROMOTER BINDING PROTEIN-LIKE7 (SPL7) (Yamasaki et al., 2009; Bernal et al., 2012) operates through microRNA 398 (miR398) (Yamasaki et al., 2007). Under Cu deficiency, SPL7 binds to GTAC motifs in the promoter regions of target genes and activates the transcription of *FSD1* as well as of *MIR398*, among other Cu-miRNAs, which in turn suppress *CSD1* and *CSD2* transcript levels. miR398 also regulates *COPPER CHAPERONE FOR SOD (CCS)* transcript levels (Sunkar and Zhu, 2004; Yamasaki et al., 2009; Beauclair et al., 2010). Additionally, under Fe deficiency, FSD1 is substituted by CSD1 and CSD2. This operates via the downregulation of miR398 under Fe deficiency, which causes the upregulation of *CSD1* and *CSD2* transcripts (Waters et al., 2012). Another so-called Cu-microRNA, miR408, is also described to participate in the metabolic adjustment to low Cu supply (Buhtz et al., 2010; Carrió-Seguí et al., 2019b). Under Cu deficiency conditions, the transcription of *MIR408* is upregulated by SPL7 via the binding to the GTAC motifs within the *MIR408* promoter (Yamasaki et al., 2009; Bernal et al., 2012; Zhang and Li, 2013). miR408 mediates the post-transcriptional downregulation of the *LAC3*, *LAC12*, and *LAC13* mRNAs encoding LACCASE-LIKE MULTICOPPER OXIDASES (LMCOs) (Abdel-Ghany and Pilon, 2008). The *A. thaliana* genome contains 17 loci encoding laccase-like multicopper oxidases (LMCOs). In all LMCO proteins the characteristic Cu binding motifs associated with MCO functionality are conserved (Supplementary Figure 1) (Turlapati et al., 2011). Laccases are glycoproteins that catalyze the oxidation *in vitro* of a wide variety of aromatic substrates including phenolic compounds and amines (McCaig et al., 2005; Turlapati et al., 2011). Based on this, LMCOs could have more diverse functions than initially expected (Reiss et al., 2013). However, their precise physiological/biochemical roles in higher plants remain largely unclear. These proteins could be involved in various physiological processes such as Cu deficiency response, Fe metabolism, lignification, and oxidative stress (McCaig et al., 2005; Turlapati et al., 2011).

**TABLE 1:** Overview of the publications reporting cross-talk between Fe and Cu homeostasis in plants.

Summary	Publication
Cu transporter *COPT2* transcript levels are upregulated by FIT under Fe-deficient conditions	Colangelo and Guerinot, 2004
Reciprocal regulation of SODs via miR398	Yamasaki et al., 2007
Role of microRNAs in the regulation of non-essential cuproproteins	Abdel-Ghany and Pilon, 2008
Fe deficiency responses include accumulation of Cu, regulation of microRNAs, and differential expression of Fe and *CuSOD* genes	Waters et al., 2012
Cu deficiency induces Fe deficiency in Arabidopsis, and this is associated with lowered root-to-shoot translocation of Fe	Bernal et al., 2012
*copt2* mutants are more tolerant to simultaneous Fe and Cu deficiency as well as more insensitive to phosphate deficiency	Perea-García et al., 2013
LPR1 and LPR2, two MCO proteins, act as ferroxidases in Arabidopsis	Müller et al., 2015
*COPT1* overexpressor rice plants show high Fe content in grains	Andrés-Bordería et al., 2017
*SPL7*-dependent repression of some components of the Fe uptake, and the ethylene response factor (ERF) redox homeostasis network	Kastoori Ramamurthy et al., 2018
Arabidopsis plants with modified miR408 levels experience de-regulation of several processes under Fe-deficient conditions	Carrió-Seguí et al., 2019b
The loss-of-function of *COPT5* induces the remobilization of the vacuolar Fe pool by inducing the expression of the Fe vacuolar transporter *NRAMP3 NRAMP4*	Carrió-Seguí et al., 2019a
A simultaneous Cu and Fe deficiency study in Arabidopsis shows a reconfiguration of large sets of molecules, such as central carbon metabolites, in particular photosynthates and amino acids	Garcia-Molina et al., 2020
Arabidopsis plants overexpressing *COPT1* or *COPT3* are not able to properly respond to Fe deficiency revealing the importance of an appropriate spatiotemporal Cu uptake for Fe homeostasis	Perea-García et al., 2020
A reduction of the fumarate synthesis in the cytosol increases the Fe availability for metalloproteins in - Cu - Fe medium	Garcia-Molina et al., 2021

Arabidopsis plants with modified miR408 levels experience the deregulation of several processes under Fe-deficient conditions, as for instance a reduction of the chlorophyll and lignin content and deregulation of the expression of several oxidative stress and lignin biosynthesis genes (Carrió-Seguí et al., 2019b). This suggests a potential role of LMCOs in Fe deficiency. However, the signaling pathway that drives a repression of miR408 levels under Fe deficiency conditions and its physiological relevance remain unknown (Carrió-Seguí et al., 2019b). *MIR408* was also identified as a target gene of ELONGATED HYPOCOTYL 5 (HY5) through the binding to G-box motifs. HY5 is a transcription factor that regulates light signaling and physically interacts with SPL7 (Zhang et al., 2014). HY5 and SPL7 can simultaneously bind to the *MIR408* promoter and coordinately control miR408 levels and its target genes in response to light and Cu, with SPL7 having a stronger impact on miR408 levels. The constitutive production of miR408 was reported to rescue the developmental phenotypes of the *hy5*, *spl7* and *hy5 spl7* mutants under Cu deficiency conditions. These results suggested that miR408 mediates light-copper crosstalk (Zhang et al., 2014). In a variety of plant species such as tobacco and rice, the overexpression of *MIR408* improves photosynthesis and growth rates as well as seed yield (Zhang et al., 2017; Pan et al., 2018; Song et al., 2018).

Another interaction between Cu and Fe homeostasis in *A. thaliana* involves the Cu TRANSPORTER (COPT) family, members of which generally mediate Cu(I) import into the cytosol (Sancenón et al., 2003). Among the *COPT* genes, COPT2 is a plasma membrane protein that functions in Cu uptake. Under Cu deficiency, *COPT2* is expressed most highly in the roots and is upregulated in dependence on *SPL7* (Yamasaki et al., 2009; Bernal et al., 2012). Additionally, COPT2 was shown to contribute to Fe homeostasis (Perea-García et al., 2013). *COPT2* transcript levels are upregulated in response to Fe deficiency and this regulation partially depends on *FER-LIKE IRON DEFICIENCY INDUCED TRANSCRIPTION FACTOR* (*FIT)*, one of the principal transcription factors that regulate the Fe deficiency response in Arabidopsis (Colangelo and Guerinot, 2004). A potential explanation for this regulation may be a prioritization of a Cu reallocation to Cu-dependent enzymes such us CSD1 and CSD2, which replace FSD1 when Fe levels are low and minimize further Fe consumption (Waters et al., 2012; Perea-García et al., 2013). The dual regulation of *COPT2* by FIT and SPL7 may explain the role of the encoded copper transporter in the interaction between the metal homeostasis of both metals. A simultaneous Cu and Fe deficiency study shows that *copt2* mutant plants are more tolerant than the wild-type plants, with an alleviation of leaf chlorosis, an increase of chlorophyll concentration and higher plastocyanin concentrations, resulting in improved growth and seed yield (Perea-García et al., 2013). In addition, based on gene expression analysis, a link is suggested between COPT2-mediated Cu uptake through the Cu transporter COPT2 and phosphate (Pi) starvation signaling, which is strongly related to Fe homeostasis (Ward et al., 2008; Perea-García et al., 2013). Transcript profiling of *copt2* and wild-type plants grown under Fe deficiency and simultaneous Fe and Cu deficiency, respectively, show that a relevant number of the upregulated transcripts are associated to Pi starvation. Under Pi starvation, the *copt2* phenotype is not affected, and the transcript levels *of COPT2* are not changed in wild-type Arabidopsis plants. Taken together, these observations suggest that *COPT2* function affects the regulation of Cu, Fe and Pi deficiency responses (Perea-García et al., 2013).

The role of other members of the COPT family in both Cu and Fe homeostasis was also studied. To analyze the interconnection between internal vacuolar pools of Fe and Cu, loss-of-function mutants of the tonoplast Cu transporter COPT5 and the Fe transporters NATURAL RESISTANCE-ASSOCIATED MACROPHAGE PROTEIN 3/4 (NRAMP3 and NRAMP4), respectively, were characterized (Carrió-Seguí et al., 2019a). This study demonstrates that the *copt5* mutant is affected by Fe deficiency and the growth of the *nramp3nramp4* double mutant is strongly damaged under Cu deficiency. *copt5* mutant plants show a strong upregulation of *NRAMP4* transcript levels whereas in the *nramp3nramp4* mutant *COPT5* transcripts are highly expressed under Cu deficiency. The authors suggested that the regulation of vacuolar Cu and Fe pools is interconnected, the absence of a functional tonoplast metal transporter for one of the metals provokes the reallocation of the other metal and the induction of its respective metal transporter (Carrió-Seguí et al., 2019a). Additionally, the overexpression of the Cu transporter *COPT1* in rice plants induces the accumulation of Fe in rice grains (Andrés-Bordería et al., 2017). Furthermore, Arabidopsis plants overexpressing *COPT1* or *COPT3* grown under Cu deficiency conditions cannot properly respond to Fe deficiency. These plants show an altered expression of several Fe homeostasis genes, including genes of the Fe uptake system and their transcriptional regulators. This suggests the relevance of a correct interconnection between Cu and Fe homeostasis under metal deficiency growth conditions (Perea-García et al., 2020).

A study addressing the role of *SPL7* in Fe homeostasis of Arabidopsis hypothesized on new potential functions for *SPL7* as a transcriptional repressor of several components of the Fe uptake machinery, such as the *IRON MAN/FEP1* (*IMA/FEP)* peptides and the subgroup Ib of *BASIC HELIX-LOOP-HELIX* (*bHLH*) transcription factor, as well as of genes of the ethylene response factor (ERF) redox homeostasis network (Kastoori Ramamurthy et al., 2018). Simultaneous Cu and Fe deficiency (-Cu -Fe) in Arabidopsis leads a reconfiguration of a huge number of molecules, such as central carbon metabolites, including a decrease of photosynthates and an increase of free amino acids under -Cu -Fe conditions. This was proposed to be associated with a swap from autotrophic to heterotrophic growth, and also includes organic acids like fumaric acid in the response to both deficiencies that may help plants to acclimate to simultaneous -Cu -Fe deficiency (Garcia-Molina et al., 2020). A more recent publication reports that the availability of cytosolic Fe is increased when the synthesis of cytosolic fumarate is decreased (Garcia-Molina et al., 2021).

In yeast and humans, Cu is essential for the uptake and distribution of Fe (Muckenthaler et al., 2008). In these organisms, the activity of MULTICOPPER OXIDASES (MCOs), which contain Cu as a cofactor, of oxidizing Fe(II) to Fe(III) is indispensable for the operation of specific transmembrane Fe transport roles (Askwith et al., 1994; Harris et al., 1995; Muckenthaler et al., 2008; Gulec and Collins, 2014). In yeast, Fe uptake involves the prior extracellular reduction of ferric chelates by the cell surface reductases FRE1 and FRE2. Subsequently, Fe^2+^ cations are reoxidized to Fe^3+^ by the multicopper oxidase FET3, which acts as a ferroxidase, and Fe^3 +^ cations are subsequently imported through the transmembrane permease FTR1. This FET3-FTR1 complex is known as the high affinity Fe uptake system. As a consequence, Cu deficiency interferes with high-affinity Fe uptake into yeast cells and can cause Fe deficiency symptoms (Askwith et al., 1994). In addition, the plasma membrane transporter FET4 mediates low-affinity Fe^2+^ uptake. Different from yeast, multicopper ferroxidases are required for cellular Fe export in humans. There are three key multicopper ferroxidases, caeruloplasmin, haephestin and zyklopen, which act in conjunction with the plasma membrane Fe^2+^ exporter ferroportin 1. Caeruloplasmin acts in physiological processes, including Cu transport and biogenic amine oxidation, and it is an essential ferroxidase in human Fe homeostasis. Haephestin is responsible for allowing the transport of dietary Fe from intestinal enterocytes into the bloodstream. Zyklopen functions to oxidize the ferrous iron to ferric iron after transfer through ferroportin from placental trophoblasts from mother to fetus (Hellman and Gitlin, 2002; Chen et al., 2010; Gulec and Collins, 2014). Analogous to the mechanism of yeast high-affinity Fe uptake, the MULTICOPPER FERROXIDASE FOX1 of the green alga *C. reinhardtii* contributes to cellular Fe acquisition under Fe deficiency (La Fontaine et al., 2002).

Bernal et al. (2012) report that severe Cu deficiency (no added Cu) induces Fe deficiency in the Arabidopsis *spl7* mutant, and that this is associated with decreased *in vitro* ferroxidase activity. Moreover, Cu deficiency causes reduced root-to-shoot Fe translocation, leading to decreased levels of the Fe storage protein ferritin and Fe-dependent catalase activity in shoots. Physiological Fe deficiency in shoots of severely Cu-deficient plants activates root Fe deficiency responses, namely increased root surface Fe(III)-chelate reductase activity and *IRT1* transcript levels, in accordance with the known systemic control of some root Fe deficiency responses. Furthermore, under Cu deficiency conditions but supplemented with extra Fe, the growth rate of the *spl7*-2 mutant shows a marked improvement, and chlorophyll concentrations are increased to ∼85% of wild-type levels. This is consistent with a model according to which, one or several Fe transmembrane transport processes of plants depend on MCO-mediated ferroxidase activity, similar to yeast, humans and green algae. Indeed, two MCO proteins, LOW PHOSPATE ROOT 1 (LPR1) and LOW PHOSPATE ROOT 2 (LPR2), together with a P5-type ATPase (PDR2), have central roles in local phosphate sensing, and this response is linked to Fe homeostasis (Svistoonoff et al., 2007; Ward et al., 2008; Ticconi et al., 2009; Müller et al., 2015). Phosphate deficiency induces the inhibition of root growth in Arabidopsis seedlings, and it is proposed that this is a consequence of Fe toxicity in the root tip (Ward et al., 2008). Interestingly, the MCO LPR1 functions as a cell-wall localized ferroxidase and is responsible for apoplastic Fe(III) deposition in roots tips under phosphate deficiency (Müller et al., 2015). Some authors hypothesize that COPT2 may be a candidate for Cu transport to LPR1 and LPR2 because roots of *copt2* mutants are longer than those of the wild-type, which is in accordance with the phenotype detected in *lpr1* and *lpr2* mutants (Svistoonoff et al., 2007; Perea-García et al., 2013). Further insights into the molecular basis of cellular Fe export pathways in plants may help to improve the knowledge of the connections between Cu and Fe homeostasis in plants, and provide important insights for combating Fe deficiency in crops and for advancing biofortification approaches.

Here, we summarize the current knowledge of the cross-talk between Cu and Fe homeostasis and provide a partial characterization of the role of Arabidopsis *LACCASE12* (*LAC12*), which encodes a member of the laccase multicopper oxidase (LMCO) protein family, in root-to-shoot Fe partitioning.

## Materials and Methods

### Plant Material

The *Arabidopsis thaliana* T-DNA insertion lines SALK_004019 (*lac12*-1, Col-0 background), SALK_047456 (*lac12*-2, Col-0 background) and SALK_070852 (*ao*, Col-0 background) were obtained from the Nottingham Arabidopsis Stock Centre, based on http://signal.salk.edu. Col-0 seeds were obtained from the NASC. The *irt1* mutant (*pam42*) was obtained from Prof. Dr. Dario Leister, LMU Munich (DE) (Varotto et al., 2002). *spl7*-2 and *frd3*-7 mutants were previously used in our lab (Bernal et al., 2012; Quintana et al., 2021).

### Growth Conditions and Experimental Treatments

For selection of homozygous mutant lines, plants were cultivated on soil. After 4 to 5 days of seed stratification on moist paper at 4°C, seeds were transferred onto pre-fertilized soil mixture Type Minitray (Balster Einheitserdewerk, Fröndenberg) in 30 × 50 cm plastic square trays. Germination and cultivation were performed in a growth chamber at 20°C, 50% relative humidity, in long-day conditions (16 h light, 8 h dark) at a light intensity of 120 μmol m^–2^ s^–1^ (Percival CU-41L4; CLF Climatics).

For sterile plant growth, seeds were rinsed in 70% (v/v) ethanol for 1 min, surface-sterilized in a solution containing 1.2% (w/v) NaOCl and 0.02% (v/v) Triton X-100 for 10 min and washed five times with ultrapure water (Milli-Q; Merck). Then, seeds were sown on a modified Hoagland’s solution containing macro- and micronutrients as described (Bernal et al., 2012), supplemented with 1% (w/v) sucrose and solidified with 1% (w/v) agar Type M (Sigma) in 12 × 12 cm square Petri plates (Greiner Bio-One). Plates were placed at 4°C in the dark for 3 d and then grown vertically for 10 days in 11 h light, 22°C/13 h dark, 18°C cycles (Percival CU-41L4; CLF Climatics). To prepare the treatment Hoagland’s medium, Agar M (Sigma) was washed with EDTA to remove all contaminant metals as described (Haydon et al., 2012). Treatment Hoagland media contained 1% (w/v) sucrose and solidified with 0.8% (w/v) EDTA-washed agar Type M (Sigma). After 10 days of growth, seedlings were transferred to treatment Hoagland media from which Fe was omitted (-Fe treatment) or added as 10 μM FeHBED (+Fe treatment) for 5 days. To perform Perls’ stain, 7-d-old seedlings were cultivated in the same conditions described above but were sown directly on the treatment media.

### Quantification of Plant Biomass and Tissue Elemental Concentrations

Shoots and roots of 15-day-old seedlings were separated with a scalpel, pooled from 20 seedlings, washed in ultrapure water and carefully blotted dry using paper towels to quantify shoot and root fresh biomass. Subsequently, shoots and roots were desorbed in 35 mL of an ice-cold solution of 5 mM CaCl_2_ and 1 mM MES-KOH, pH 5.7, for 10 min, in ice-cold solution of 5 mM CaSO_4_, 10 mM Na_2_EDTA, pH 5.7 for 5 min, and twice in ice-cold ultrapure water for 1 min. Shoot and root tissues were dried at 60°C for 3 d and equilibrated at room temperature for at least 3 d before homogenization. Dried shoot and root material was homogenized, weighed, and subsequently digested for the quantification of elemental concentrations by inductively coupled plasma atomic emission spectrometry (ICP-OES) in an iCAP 6500 DUO instrument (Thermo-Fisher) as described previously (Sinclair et al., 2017). Three independent experiments were carried out, each comprising 4 to 5 plates per genotype and treatment.

### Genotyping

Plants homozygous for the *lac12* T-DNA insertions were identified by PCR on genomic DNA: The specific primer for the left border of the T-DNA (5′-GCGTGGACCGCTTGC TGCAACT-3′) was used in combination with a *LAC12* specific primer (5′- CACCATGACGACTGTTCACACATTCTCT-3′) for SALK_004019 (*lac12*-1) and for SALK_047456 (*lac12*-2), respectively. *LAC12* WT allele was identified by PCR on genomic DNA using the following *LAC12* specific primers, LAC12_F 5′-CACCATGACGACTGTTCACACATTCTCT-3′ and LAC12-R 5′-CTAGCAAATAGGTAGATCGTGAGGA-3′. Plants homozygous for the *ao* T-DNA insertions were identified by PCR on genomic DNA: The specific primer for the left border of the T-DNA (5′-GCGTGGACCGCTTGCTGCAACT-3′) was used in combination with an *AO* specific primer (5′- CACCATGATGAGACCGAAGAGATCA -3′) for SALK_070852. *AO* WT allele was identified by PCR on genomic DNA using the following *AO* specific primers, AO-F 5′- CACCATGATGAGACCGAAGAGATCA -3′ and AO-R 5′-TCAGCGTTTAGTCTGACCACATCC -3′. Genomic DNA isolation was done as described (Edwards et al., 1991).

### RNA Extraction, cDNA Synthesis, and Real-Time PCR

Total RNA was isolated from 100-mg subsamples of frozen root and shoot tissues exactly as described (Bernal et al., 2012). Quality and quantity of RNA was analyzed spectrophotometrically at the wavelengths of 260 and 280 nm, respectively. Equal amounts of RNA (1 μg) were used as a template for cDNA synthesis (RevertAid First Strand cDNA Synthesis Kit, Thermo Fisher Scientific) with oligo-dT primers following manufacturer′s instructions. RT-qPCR was performed on a LightCycler480 (Roche) in a 10-μL reaction mixture containing 16 ng of cDNA, each primer at 0.25 μM and 5 μL of 2X GoTaq qPCR Master Mix (Promega) as described (Quintana et al., 2021). Reaction efficiencies (RE) for each PCR reaction were determined with the LinRegPCR program, version 2016.0 (Ruijter et al., 2009). Threshold cycle values were calculated for each reaction at a threshold value of the normalized reporter *R*_n_ of 0.2. Relative transcript levels were calculated as follows: RTL = 1000 × RE_m_^–Δ*CT*^, whereby RE_m_ is the arithmetic mean of the reaction efficiency and ΔC_T_ values were calculated as follows: ΔC_*T*_ = C_T_ (target gene) – C_T_ (constitutively expressed reference gene: *UBQ10*). Primers sequences are listed in Supplementary Table 1.

### Yeast Constructs, Strains, and Growth

*IRT1* and *LAC12* cDNAs, including translational start and stop codon, were amplified from wild-type cDNA by RT-PCR with IRT1-ATG_F 5′-CACCATGGCTTCAAATTCAGCAC-3′ and IRT1-cDNA_TAA_R 5′-TTAAGCCCATTTGGCGATAATCG-3′ and with LAC12-ATG_F 5′-CACCATGACGACTGTTCACACA TTCTCT-3′; LAC12-TAG-R 5′-CTAGCAAATAGGTAGATC GTGAGGA-3, respectively. Both cDNAs were cloned into pENTR-D/TOPO (Invitrogen) and verified by Sanger sequencing before performing the recombination reaction into the yeast expression vector pFL61-Gateway (Desbrosses-Fonrouge et al., 2005) using LR clonase (Invitrogen) according to manufacturer′s protocol. Both constructs were checked by Sanger sequencing. For complementation assays, competent *S. cerevisiae* yeast cells of the wild-type (WT, DY1457) and *fet3fet4* mutant (DEY1453; Dix et al., 1994) strains were transformed with 1 μg of each construct or the empty vector (Dohmen et al., 1991). The corresponding WT yeast strain transformed with the empty vector was used as positive control. Transformants were selected on SD media lacking uracil (SD-Ura) pH 5.7 with 2% (w/v) D-glucose as a carbon source. For each construct, three independent transformant colonies were grown overnight at 30°C in 2 mL SD-Ura pH 5.7 media to early stationary phase (OD_600_ ≈ 0.3, *ca*. 10^7^ cells ml^–1^). Yeast cells were then centrifuged at 13.000 rpm for 1 min, washed once with SD-Ura pH 5.7 media with 2% (w/v) D-glucose and resuspended in the same media. Aliquots of cell suspensions were serially diluted with SD-Ura pH 5.7 media with 2% (w/v) D-glucose in a 10-fold series (*ca*. 10^7^, 10^6^, 10^5^, and 10^4^ cells ml^–1^, based on OD_600_). Drops of 10 μL were spotted onto solid (2% w/v Bacto agar, Difco) SD-Ura pH 5.7 media with 2% (w/v) D-glucose supplemented with or without 0.5 mM FeSO_4_. Plates were incubated at 30°C for 3 days and photographed using a Nikon Digital SLR camera with an AF-S DX Zoom NIKKOR 18–70 mm 1:3, 5–4,5G ED-IF objective.

### Localization of Fe(III) With Perls’ Stain

7-d-old seedlings germinated and grown on Fe-sufficient (+Fe, 10 μM FeHBED) or Fe-deficient (-Fe, 0 μM FeHBED) Hoagland’s medium solidified with EDTA-washed agar were harvested at ZT1. Roots (∼4 cm from root tip) of 3 to 5 seedlings were pooled and washed once with ice-cold 10 mM EDTA (pH 5.7) for 5 min and three times with ice-cold ultrapure water for 1 min. Then, the samples were vacuum-infiltrated with Perls’ stain solution (equal volumes of 4% (v/v) HCl and 4% (w/v) potassium ferrocyanide) for 15 min (Roschzttardtz et al., 2009). Roots were incubated in the Perls’ stain solution for another 15 min at room temperature and then rinsed three times with ultrapure water and mounted in ultrapure water for visualization. DAB intensification was performed as described (Brumbarova and Ivanov, 2014). Photographs were taken using an Axioscope microscope, Axiovision sofware (Zeiss) and Axiocam MRc Rev. 3 camera.

### *In-gel* Detection of Ferroxidase and Phenoloxidase Activities

These assays were performed exactly as described (Bernal et al., 2012). The experiment was repeated twice using independent protein samples extracted from aliquots of frozen homogenized tissues harvested in a common experiment. Band quantification was done using the ImageJ software.

### Measurement of Chlorophyll Concentrations

Total chlorophyll was extracted with 1.5 ml methanol from 20 to 40 mg of frozen ground shoot tissues of 15-day-old seedlings. Absorbance values were determined spectrophotometrically at the wavelengths of 652 and 665 nm in 96-well plates in a Synergy HTX Multi-Mode Reader (Agilent, former BioTek), using methanol as a blank. Microplate path length-correction to 1 cm was carried out with the factor 0.51 according to Warren, 2008. Chlorophyll concentrations were calculated as described (Porra et al., 1989) and normalized to fresh biomass.

### *In silico* Sequence Analysis of MCO in *Arabidopsis thaliana*

Search of the TAIR10 protein databases was done with the BLAST programme (Altschul and Lipman, 1990) using the previously identified *Saccharomyces cerevisiae* FET3 protein sequence, the multicopper ferroxidase (MCO) involved in high-affinity Fe uptake. Alignments were done using Mafft program (Katoh et al., 2002). The location of putative transmembrane helices was predicted using the programme ARAMEMNON at http://aramemnon.botanik.uni-koelm.de/ (Schwacke et al., 2003).

### 3D-Structure Modeling

To model the 3D-structure of LAC12, LAC13 and AO from *Arabidopsis thaliana*, the Phyre2 version 2.0 was used (Kelley and Sternberg, 2009) and subsequently compared with the well-known 3D-structure of ScFET3 (Taylor et al., 2005). LAC12, LAC13 and AO 3D-structure models were submitted to the 3DLigandSite server to predict *in silico* metal binding (Wass et al., 2010).

### Statistical Analysis

Multiple comparisons were conducted by two-way analysis of variance (ANOVA; Tukey’s honestly significant difference [HSD]) using Statgraphics software (version XV.I; Statpoint Technologies).

## Results

### *In silico* Identification of FET3 Homologs in *Arabidopsis thaliana*

In humans, membrane-associated caeruloplasmin, a multi-copper oxidase, drives Fe export from cells *via* the Fe^2+^ exporter ferroportin by oxidizing Fe^2+^ upon its arrival on the external face of the plasma membrane (Hellman and Gitlin, 2002). Previously, we observed that severely Cu-deficient plants are also Fe-deficient as a result of impaired root-to-shoot Fe partitioning (Bernal et al., 2012). We speculated on a possible role of a MCO in the cellular export of Fe into xylem vessels in roots of *A. thaliana*, in analogy with membrane-associated human caeruloplasmin. Therefore, we conducted an *in silico* analysis to identify candidate MCOs functioning as ferroxidases based on the best studied MCO FET3 of the high-affinity Fe uptake system of *Saccharomyces cerevisiae* (Askwith et al., 1994). FET3 has four catalytic Cu ions in the mononuclear Cu cluster (T1) and the trinuclear Cu cluster (T2/T3), where the oxidation of Fe^2+^ cations takes place. Based on site-directed mutagenesis of the *S. cerevisae* FET3 protein, previous research identified D319 and D320 to be critical for the growth of yeast under Fe-deficient conditions. In particular, mutation of D320 abrogated the FET3-dependent Fe uptake function (Bonaccorsi di Patti et al., 2005; Quintanar et al., 2007). Kinetic studies of mutations of the E185, D283, and D409 residues, respectively, showed their important role in Fe(II) oxidation (Quintanar et al., 2007). In LPR1 and LPR2, two MCOs that act as ferroxidases mediating root phosphate deficiency responses in Arabidopsis, the acidic residues of FET3 are also conserved (E269, D370 and D462 in LPR1 and E271, D372 and D464 in LPR2) as shown the structural superimpositions of LPR1 and LPR2 onto FET3, respectively (Müller et al., 2015). As a first step, a BLAST search using the yeast FET3 sequence generated a list of candidate MCOs that may have ferroxidase functions in *A. thaliana*, among them the LMCO family and several ascorbate oxidases (Table 2). An alignment of the LMCO, the ascorbate oxidase AO and the LPR1 and LPR2 sequences from *A. thaliana* with the sequence of FET3 from yeast revealed the conserved C and H residues that form the trinuclear (T2/T3) and mononuclear (T1) Cu binding sites (Supplementary Figure 1). The essential ferroxidase E185, D283, and D409 residues of yeast FET3, seem to be conserved in LAC3 (E219, D333, D462), LAC5 (E220, D341, D472) LAC12 (E218, D326, D457), LAC13 (E217, D331, D461), LPR1 (E269, D370, and D462), LPR2 (E271, D372, and D464) and AO (E198, D354, and D465) (Supplementary Figure 1, residues highlighted in pink), and the ferroxidase aspartic residues D319 and D320 of FET3 appear to be conserved in LAC8 (D407, D408), LAC9 (D410, D411), LAC12 (D351, D352) and LAC16 (D367, D368) (Supplementary Figure 1, residues highlighted in red). This conservation was suggested based on our BLAST results and alignment, as well as on the comparison of the 3D-structure of FET3 with the 3D-structure models of LAC12, LAC13, and AO (Supplementary Figure 2A). Our *in silico* analysis was also compared with the homology modeling previously conducted for LPR1 and LPR2 and the structural superimpositions of LPR1 and LPR2 onto FET3 that allowed to assign the ferroxidase residues in Arabidopsis (Müller et al., 2015). The two laccase multicopper oxidase proteins (LMCOs) LAC12 (At5g05390) and LAC13 (At5g07130), and AO (At4g39830), a MCO-ascorbate oxidase of the cupredoxin superfamily, were among the Arabidopsis proteins showing highest similarity (42%, 41%, and 43%, respectively) to FET3 (Table 2). In addition, LAC12 and AO also contained one predicted transmembrane domain as FET3 (Supplementary Figures 1, 2B) (McCaig et al., 2005; Turlapati et al., 2011; Santamaría et al., 2018). According to the eFP browser, *LAC12* and *LAC13* were expressed mainly in the vasculature of the root maturation zone adjacent to the xylem (Brady et al., 2007; Winter et al., 2007) (Supplementary Figure 3A).

**TABLE 2:** Putative candidate genes for ferroxidase functions identified through *in silico* analysis.

AGI code	Name	Description	TM domain	Fe-uptake-like D residues^1^	% Homology with *Sc*FET3	Publication
AT5G21105	None	Plant L-ascorbate oxidase	1	Yes	41	Unpublished
AT4G39830	Ascorbate oxidase AO	Cupredoxin superfamily protein	1	Yes	43	Santamaría et al., 2018
AT5G21100	None	Plant L-ascorbate oxidase	1	Yes	42	Unknown
AT2G30210	Laccase 3 (LAC3)	Member of laccase family of genes	1	Yes	42	Rojas-Murcia et al., 2020; Zhuang et al., 2020
AT5G05390	Laccase 12 (LAC12)	Member of laccase family of genes	1	Yes	42	Carrió-Seguí et al., 2019b
AT5G07130	Laccase 13 (LAC13)	Member of laccase family of genes	1	Yes	41	Carrió-Seguí et al., 2019b; Rojas-Murcia et al., 2020

*^1^*S. cerevisiae* ferroxidase FET3 (Askwith et al., 1994) contains essential aspartic residues which play an important role in Fe(II) oxidation (Bonaccorsi di Patti et al., 2005; Quintanar et al., 2007).*

### *LAC12* Is Upregulated Under Fe Deficiency in Wild-Type Seedlings

**FIGURE 1:**
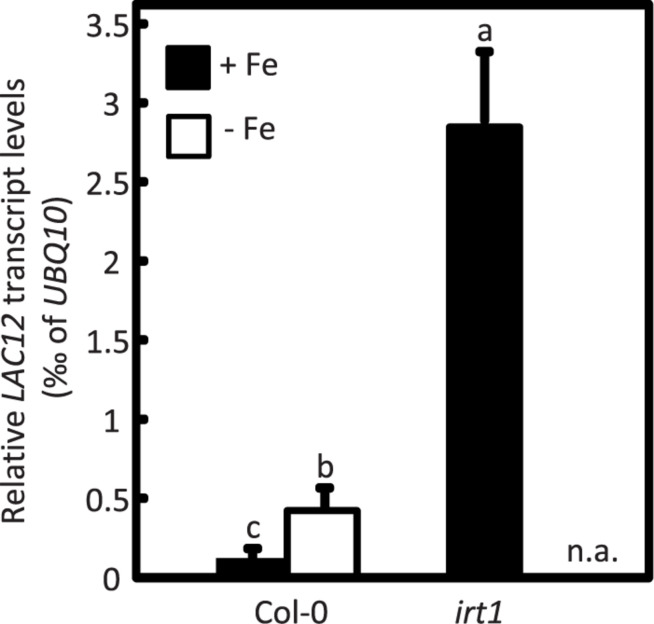
*LAC12* transcript levels increase under Fe deficiency. Relative *LAC12* transcript levels in roots of wild-type (Col-0) and *irt1* seedlings are analyzed by RT-qPCR. Seedlings were cultivated in standard Hoagland’s medium for 10 d (5 μM FeHBED; unwashed agar) followed by a growth period of 5 d on Fe-sufficient (+Fe, 10 μM FeHBED) and Fe-deficient (-Fe, 0 μM FeHBED) agar-solidified Hoagland’s medium (EDTA-washed agar), on vertically oriented petri plates. Bars show arithmetic means ± SD of relative transcript levels normalized to *UBQ10*, and were calculated from *n* = 3 technical replicates from one experiment representative of four independent biological experiments. n.a. not analyzed. Different characters denote statistically significant differences (*P* < 0.05) between means based on ANOVA (Tukey’s HSD).

To examine if *LAC12* expression was regulated under Fe deficiency, we cultivated wild-type (WT) and *irt1* seedlings under Fe-deficient and Fe-sufficient (control) conditions for 10-d, subsequent to a 5-d pre-cultivation period on Fe-sufficient medium (Figure 1; Quintana et al., 2021). The *IRON REGULATED TRANSPORTER1* (*irt1*) mutant defective in root high-affinity Fe uptake was included as a control genotype exhibiting constitutive activation of the Fe deficiency responses (Connolly et al., 2002; Henriques et al., 2002; Varotto et al., 2002; Vert et al., 2002; Kobayashi and Nishizawa, 2012). Root *LAC12* transcript levels were 3.5 to 4-fold elevated under Fe deficiency conditions compared to control conditions in the wild-type, and 24-fold elevated in the *irt1* mutant. Taking into account these results, we decided to characterize the multi-copper oxidase (MCOs) *LAC12* as a candidate ferroxidase.

### A *LAC12* cDNA Complements the Fe Uptake-Defective Yeast *fet3fet4* Mutant

**FIGURE 2:**
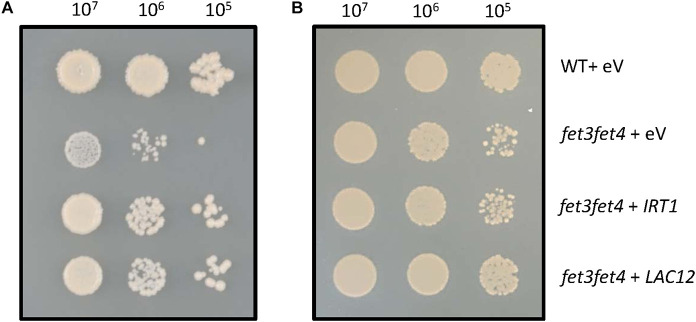
Heterologous expression of the *AtLAC12* cDNA complements a Fe uptake-defective *fet3fet4* mutant of *Saccharomyces cerevisiae*. Wild-type and the Fe uptake-defective *fet3fet4* mutant of *Saccharomyces cerevisiae* transformed with the empty vector pFL61 Gateway (eV) or expressing *IRT1* or *LAC12* cDNAs of *A. thaliana*, respectively. Aliquots of 10 μL of 10-fold serial dilutions (starting from OD_600_ = 0.3, *ca*. 10^7^ cells ml^–1^) were spotted on **(A)** SD-URA medium (pH 5.7) and **(B)** SD-URA medium (pH 5.7) supplemented with 0.5 mM FeSO_4_. Images are representative of three independent transformant colonies from each of two independent experiments.

We tested whether heterologous expression of the *LAC12* coding sequence complements the Fe uptake-defective *S. cerevisiae fet3fet4* mutant (Figure 2). Expression of *LAC12* significantly improved *fet3fet4* mutant growth compared with the mutant strain transformed with empty vector on ordinary SD medium (Figure 2A), similar to yeast *fet3fet4* cells expressing the *IRT1* cDNA as a positive control (Eide et al., 1996). Both the *fet3fet4* mutant and all yeast transformants were able to grow equally well on medium supplemented with 0.5 mM Fe (Figure 2B). This result suggests that in yeast, LAC12 may be able to function as a multicopper oxidase with ferroxidase activity, which would be sufficient to complement *fet3fet4*.

### Arabidopsis *lac12* Mutants Are Sensitive to Fe Deficiency

**FIGURE 3:**
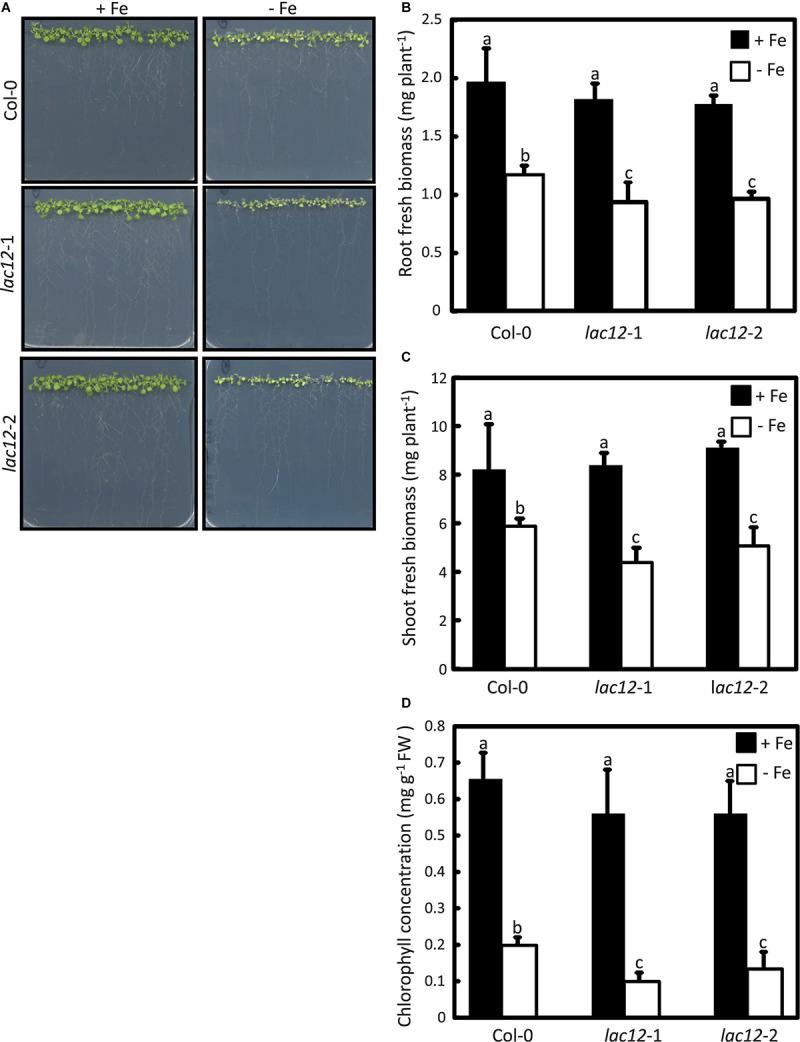
Arabidopsis *lac12* mutants are more sensitive to Fe deficiency than the wild-type. **(A)** Photographs of 15-day-old seedlings of WT, *lac12-*1 and *lac12-*2 mutant lines grown in standard Hoagland’s medium for 10 d (5 μM FeHBED; unwashed agar) followed by a growth period of 5 d on Fe-sufficient (+Fe, 10 μM FeHBED) and Fe-deficient (-Fe, 0 μM FeHBED) agar-solidified Hoagland’s medium (EDTA-washed agar), on vertically oriented petri plates. Photographs are from one experiment representative of three independent experiments. Fresh biomass of **(B)** roots and **(C)** shoots, and **(B)** leaf chlorophyll content of WT, *lac12*-1 and l*ac12*-2 seedlings grown on vertically oriented petri plates as described above. Bars show arithmetic means ± SD of three replicate plates, with 20 seedlings per plate, from one experiment representative of three independent experiments. Each replicate consisted of pooled material of 20 seedlings from one plate **(B** and **C)**, or 5 seedlings per replicate plate **(D)**. Different characters denote statistically significant differences (*P* < 0.05) between means based on ANOVA (Tukey’s HSD).

To further investigate the role of *A. thaliana LAC12*, two independent T-DNA insertion lines disrrupted in the *LAC12* gene were identified in the Columbia-0 (Col-0) background and characterized (see Materials and Methods). The T-DNAs insertions were confirmed in the 4th exon (*lac12*-1) and 5th exon (*lac12*-2) of the *LAC12* gene, respectively (Supplementary Figures 3B,C). *LAC12* transcript levels were reduced in both *lac12* T-DNA insertion lines (Supplementary Figure 3E). To examine if Fe deficiency affects the growth of *lac12* mutant seedlings, wild-type and *lac12* mutant seedlings were cultivated in Fe-deficient and Fe-sufficient agar-solidified media (Haydon et al., 2012). Under Fe-sufficient conditions, the appearance, biomass and leaf chlorophyll concentrations were similar in wild-type seedlings and the two *lac12* mutant lines (Figure 3). On Fe-deficient media, characteristic symptoms of Fe deficiency were detected in the wild-type (Marschner, 1995) including chlorosis, reduced root and shoot biomass and decreased leaf chlorophyll concentrations. Compared to wild-type seedlings, both *lac12* mutant lines were more sensitive to Fe deficiency, with more severely reduced biomass and chlorophyll concentrations (Figure 3). These data suggest that the multicopper oxidase *LAC12* is required for wild-type levels of plant growth under Fe deficiency. This means that LAC12 function seems to be necessary to allow plants to behave as wild-type plants under Fe deficiency.

### Elevated Root Fe Accumulation and Decreased Shoot Total Fe in Arabidopsis *lac12* Mutants

**FIGURE 4:**
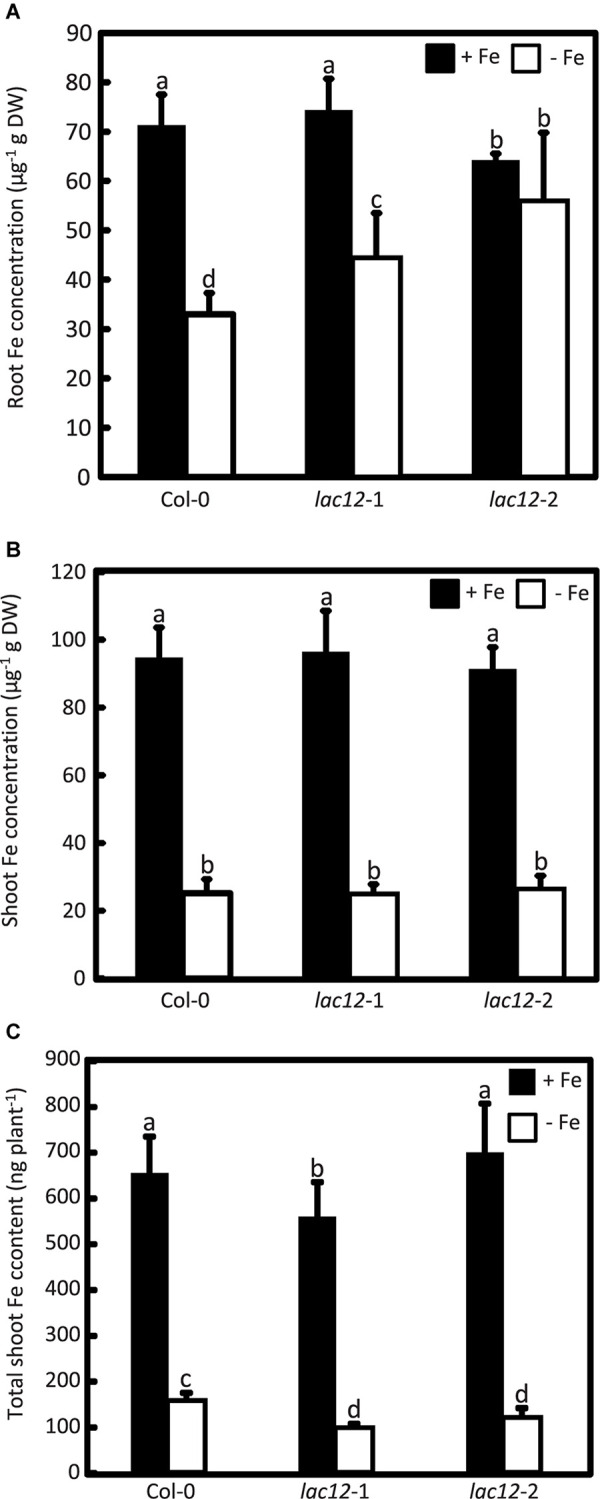
Arabidopsis *lac12* mutants accumulate higher Fe concentrations in roots and contain lower total shoot Fe than the wild-type when cultivated under Fe deficiency. Fe concentrations in **(A)** roots and **(B)** shoots, and **(C)** total Fe content in shoots, of WT, *lac12*-1 and *lac12*-2 seedlings grown on vertically oriented petri plates as described in Figure 3. Values are arithmetic means ± SD of three biological replicate plates from one experiment representative of three independent biological experiments. Metals were quantified in pooled tissues from 15 seedlings per replicate plate. Different characters denote statistically significant differences (*P* < 0.05) between genotypes and Fe treatments based on ANOVA (Tukey’s HSD).

To test whether Fe levels in plant tissue are altered alongside the enhanced sensitivity to Fe deficiency of *lac12* mutants, we quantified metal concentrations by inductively coupled plasma optical emission spectrometry (ICP-OES) in shoots and roots of seedlings cultivated in Fe-sufficient and Fe-deficient agar-solidified media (Figure 4). Upon cultivation in control conditions, root (Figure 4A) and shoot (Figure 4B) Fe concentrations in wild-type and *lac12* mutant seedlings were mostly equivalent, except for *lac12*-2 in which Fe concentrations in roots were lower compared to wild-type and *lac12*-1, respectively. However, on Fe-deficient media, root Fe concentrations of *lac12* mutants were between 20% (*lac12*-1) and 30% (*lac12*-2) elevated compared to wild-type seedlings. Total shoot Fe content was between 40% (*lac12*-1) and 25% (*lac12*-2) lower in *lac12* mutants than in the wild-type under Fe deficiency conditions (Figure 4C). Shoot Fe concentrations of these plants were very low and similar between wild-type and *lac12* mutants, consistent with growth limitation by Fe.

**FIGURE 5:**
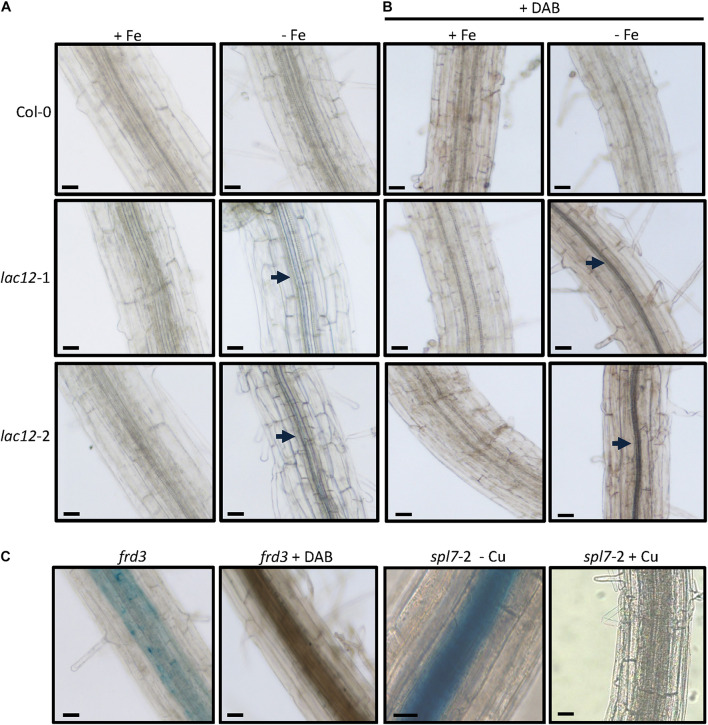
Fe localization in roots of *lac12* mutants. **(A,B)** Histochemical detection of Fe(III) by Perls’ blue stain in the root maturation zone of 7-d-old WT, *lac12*-1 and *lac12*-2 seedlings grown on Fe-sufficient (+Fe, 10 μM FeHBED) or Fe-deficient (-Fe, 0 μM FeHBED) agar-solidified Hoagland’s medium (EDTA-washed agar) without **(A)** or with DAB intensification **(B)**. **(C)** Histochemical detection of Fe(III) by Perls’ blue stain in the root maturation zone of *frd3* and *spl7-*2 seedlings. The *frd3* mutant seedlings were grown only under Fe-sufficient conditions for 7 days. Images are of 21-d-old *spl7*-2 seedlings grown on vertical glass plates containing EDTA-washed agar-solidified Hoagland’s medium (0.5 μM CuSO_4_, control) or the same medium containing no added copper (-Cu) as described (Bernal et al., 2012). Photographs are representative of *n* = 10 to 12 roots stained and imaged in one experiment. Photographs are from one experiment representative of two independent experiments. Arrows point to Perls’ stain-positive regions. Scale bars: 50 μm.

Increased Fe concentrations in roots of *lac12* mutant seedlings compared to wild-type roots upon cultivation under Fe deficiency prompted us to use Perls’ stain for Fe localization, without and with DAB intensification (Figures 5A,B and Supplementary Figure 5). Perls’ stain detected Fe in the vascular tissue in *lac12* mutant roots grown under Fe deficiency, but not in wild-type roots (Figures 5A,B). The signal was weak and appeared to be present primarily in the stele, this result was additionally supported by combining Perls’ stain with the DAB intensification step (Figure 5B). Upon cultivation in control conditions, no Perls’ stain was detected in either wild-type nor *lac12* mutants. This result is counter-intuitive because in all lines analyzed the Fe concentrations in control conditions were higher than in Fe deficient conditions. A possible explanation may lie in a different Fe distribution across the root, whereby Fe may usually be widely distributed among the entire root and thus not detectable by Perls’ stain, with a more highly localized Fe accumulation in *lac12* mutant seedlings grown under Fe-deficient conditions. This result is in full agreement with the strong Fe localization observed in the stele in *frd3-*7 and s*pl7*-2 mutants, in which root-to-shoot Fe partitioning is impaired (Figure 5C) (Green and Rogers, 2004; Bernal et al., 2012). Taken together, our data suggest that the disruption of *LAC12* interferes with root-to-shoot Fe partitioning under Fe deficiency.

Irrespective of their genotype, Cu concentrations were higher in plants grown under Fe deficiency compared to Fe-sufficient conditions (Supplementary Figure 6), as previously reported (Waters et al., 2012). Root Mn concentration of wild-type and *lac12* mutant plants are higher compared to shoot Mn concentrations in Fe-sufficient conditions (Supplementary Figure 6). Under Fe deficiency, Mn concentrations in both roots and shoots of wild-type and *lac12* mutant plants are higher than in Fe-sufficient conditions, in which the increase of root Mn concentration in *lac12* mutants were significantly higher when compared to wild-type plants. This result could be related to an increase of IRT1 protein levels, which is well known to transport Mn as well (Eide et al., 1996; Rogers et al., 2000), as is known to happen in Fe-deficient wild-type plants (Quintana et al., 2021). The accumulation of Zn under Fe deficiency has been previously reported in WT plants (Buckhout et al., 2009) and could be due to a low specificity of IRT1, which is transcriptionally induced under Fe-deficient conditions (Supplementary Figure 6) (Vert et al., 2002).

### Decreased Ferroxidase and Phenoloxidase Activities in *lac12* Mutant Plants

**FIGURE 6:**
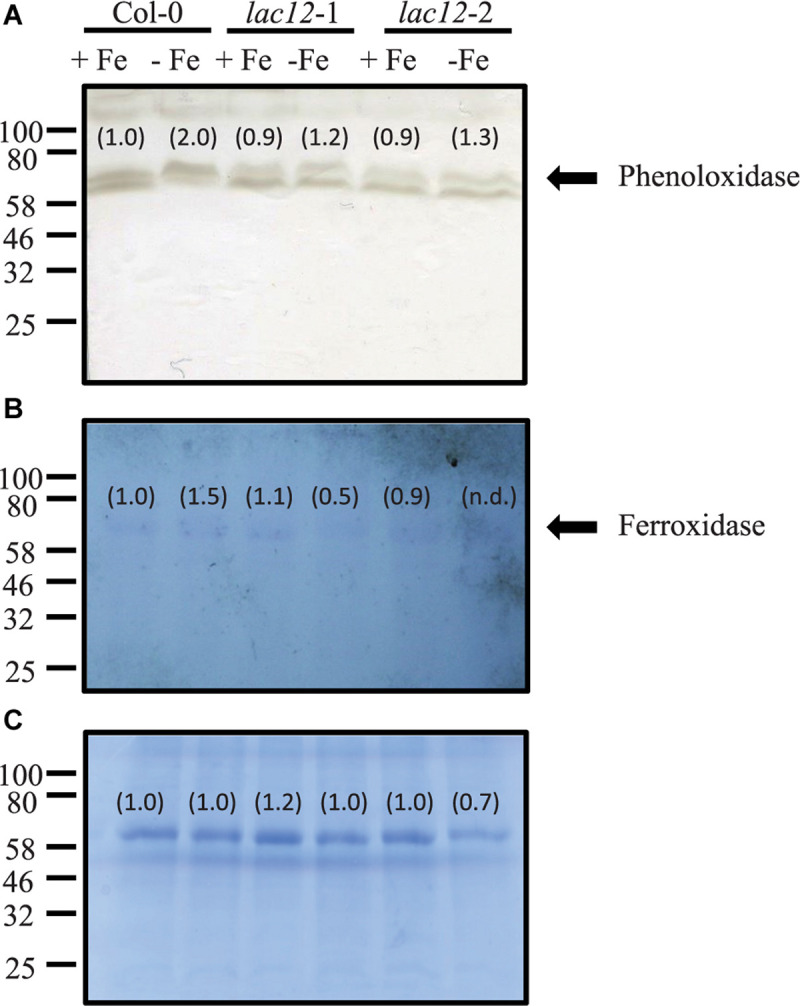
*In-gel* phenoloxidase and ferroxidase activities in *lac12* mutants. *In-gel* detection of phenoloxidase **(A)** and ferroxidase **(B)** activities in total protein extracts from roots of WT, *lac12*-1 and *lac12*-2 seedlings grown on vertically oriented petri plates as described in Figure 3. Thirty micrograms of protein were loaded per lane. Data shown are from one single independent experiment. **(C)** Coomassie blue staining to visualize protein loading.

The functional complementation of the *fet3fet4* yeast double mutant with *LAC12* (Figure 2) prompted us to further analyze the existence of MCO-related enzyme activities in WT and *lac12* mutant plants and its regulation by Fe deficiency (Figure 6). We observed that phenoloxidase and ferroxidase activities in root crude extracts run similarly in denaturing SDS-PAGE protein gels, suggesting that one or several MCO proteins may have, additionally, ferroxidase activity. It is worth mentioning at this point that the *in-gel* phenoloxidase activity assay detected two bands of activity in all root samples tested, however, we only discuss further the abundance of the upper band because this is the one that runs at a similar position as the band exhibiting ferroxidase activity. Wild-type roots showed an increase in phenoloxidase activity under Fe-deficient conditions. In roots of *lac12* mutants, this phenoloxidase activity was lower than in wild-type under both Fe-sufficient conditions and Fe-deficient conditions (Figure 6A). Ferroxidase activity was detectable in all root samples and increased slightly under Fe-deficient conditions in wild-type roots (Figure 6B). In *lac12* roots, ferroxidase activities were similar to wild-type roots under Fe-sufficient conditions, but decreased under Fe-deficient conditions (Figure 6A). Taken together, under Fe deficiency when *LAC12* transcript levels are elevated in the wild-type, decreased activities of both phenoloxidase and ferroxidase in *lac12* mutants suggest that LAC12 can act as a ferroxidase in Arabidopsis roots.

### Characterization of the Fe Deficiency Responses in *lac12* Plants

**FIGURE 7:**
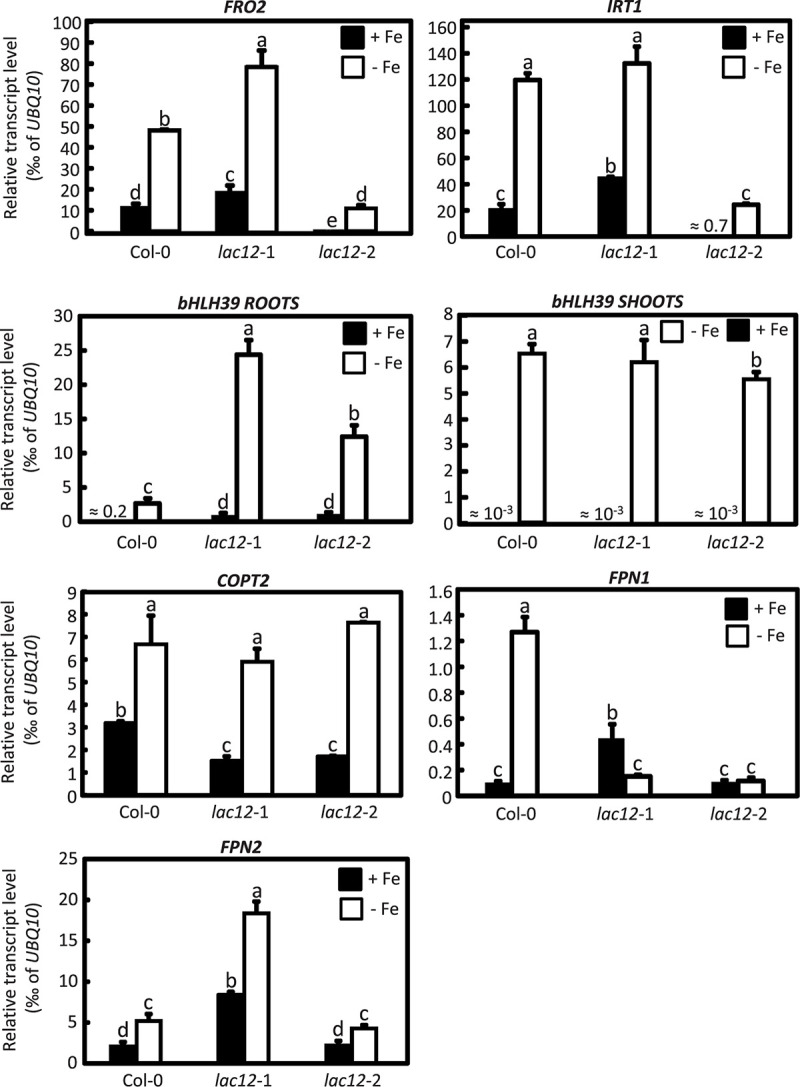
Real time RT-qPCR quantification of several Fe-deficiency responsive transcripts in *lac12* mutants. Relative transcript levels of *FRO2, IRT1, bHLH39, COPT2, FPN1*, and *FPN2* in roots and *bHLH39* in shoots of WT, *lac12*-1 and *lac12*-2 seedlings grown on vertically oriented petri plates as described in Figure 3, analyzed by RT-qPCR. Bars show arithmetic means ± SD of relative transcript levels normalized to *UBQ10*, and were calculated from *n* = 4 technical replicates from one experiment. Different characters denote statistically significant differences (*P* < 0.05) between means based on ANOVA (Tukey’s HSD).

To further examine Fe status in *lac12* mutants, preliminary data regarding the regulation of the transcript levels of a subset of well-known Fe deficiency response genes were obtained by RT-qPCR in the wild-type and *lac12* mutants grown under Fe-sufficient and Fe-deficient conditions (Figure 7). *FRO2* and *IRT1* transcript levels were strongly increased in roots and *bHLH39* was strongly upregulated in roots and shoots under Fe-deficient conditions in both wild-type and *lac12* mutant plants. The expression of *bHLH39* in Fe-deficient *lac12* roots was higher than in Fe-deficient wild-type roots. This may indicate that the expression of some genes of the Fe deficiency response machinery is enhanced in the *lac12* mutant plants compared to wild-type plants under Fe-deficient conditions. Additionally, *COTP2*, a known Fe-deficiency responsive transcript (Colangelo and Guerinot, 2004) encoding a Cu transporter, was upregulated in both wild-type and *lac12* mutant plants under Fe-deficient conditions. Transcript levels of *IRON REGULATED 1*/*FERROPORTIN 1* and *2* (*IREG1*/*FPN1, IREG2/FPN2*) were both increased in Fe-deficient wild-type roots compared to controls (Figure 7).

### Arabidopsis *ao* Mutants Are Sensitive to Fe Deficiency

To elucidate whether other genes may account for the Cu requirement in plant Fe homeostasis, a preliminary characterization of the *ASCORBATE OXIDASE (AO)* gene function, another promising candidate, was conducted. One T-DNA insertion line disrupted in the *AO* gene was isolated in the Columbia-0 (Col-0) background and characterized (see Materials and Methods). The T-DNA insertion was confirmed in the 3rd exon of the *AO* gene (Supplementary Figures 3B,D). To investigate if the growth of the *ao* mutant line is affected under Fe deficiency, the same physiological experiments were carried out as for *lac12* mutants (Supplementary Figure 4). The *ao* mutant seedlings produced less root and shoot biomass and contained lower chlorophyll concentrations (Supplementary Figures 4A–D). Under Fe deficiency, shoot Fe concentrations were similar in the *ao* mutant and the wild-type (Supplementary Figure 4E). The data for root Fe concentrations were not reliable because of insufficient biomass obtained. Cu, Zn and Mn concentrations were elevated in wild-type and *ao* mutant seedlings grown under Fe-deficient compared to Fe-sufficient conditions (Supplementary Figure 6). Future work will be required to ascertain the relevance of *AO* in *A. thaliana* under Fe deficiency.

## Discussion

Less than a decade ago, researchers began to address the interactions between Cu and Fe homeostasis in plants, including their uptake by roots, storage and movement within the plant as well as regulatory processes and the physiological relevance of possible interactions at the cellular and whole-plant levels. One goal of this report was to highlight recent progress made on the cross-talk among the homeostasis of Fe and Cu (for more details see Table 1 and Introduction section). To expand this further, we also report here initial data toward a characterization of the roles of the two genes *LAC12* and *AO* encoding multicopper oxidases (MCO), in the partitioning of Fe between roots and shoots in *A. thaliana*.

Previously, we observed that severe Cu deficiency causes Fe deficiency apparently as a result of an impairment of root-to-shoot Fe translocation, and this was associated with reduced *in vitro* ferroxidase activity (Bernal et al., 2012). We hypothesized on a role of one or several MCOs in enabling a possible cellular export step of Fe into the xylem for root-to-shoot Fe translocation in *A. thaliana*, similar to what is known on the importance of ferroxidases in the cellular export of Fe in humans (Bernal et al., 2012). A comparison of the structural superimpositions of LPR1 and LPR2 onto FET3, which allowed to assign the ferroxidase residues in Arabidopsis (Müller et al., 2015) with a BLAST search, alignment and 3D-structure model of LAC12, LAC13 and AO (Table 2 and Supplementary Figures 1, 2A), suggested that LAC3, LAC5, LAC12, LAC13, and AO conserved the essential ferroxidase E185, D283, and D409 residues from yeast as well as the MCO ferroxidases LPR1 and LPR2 (more details on conserved residue positioning in results) (Müller et al., 2015). Among these proteins, LAC12, LAC13 and AO showed the highest homology to FET3. As a first step toward elucidating the roles of LMCO proteins in Fe homeostasis of *A. thaliana*, we focused here primarily on LAC12.

We observed that *LAC12* transcript levels were upregulated under Fe deficiency in wild-type plants compared to control conditions (Figure 1) similar to well-known Fe deficiency responsive genes (Figure 7). This result was in agreement with previously published data (Carrió-Seguí et al., 2019b). These authors used plants with modified miR408 levels, a miRNA that mediates the post-transcriptional downregulation of *LAC3*, *LAC12*, and *LAC13* mRNAs (Abdel-Ghany and Pilon, 2008). They found that under Fe deficiency conditions, all the studied miR408 targets, such as *LAC12* and *LAC13*, were upregulated whereas mir408 was downregulated, in wild-type plants (Carrió-Seguí et al., 2019b). Surprisingly, phenoloxidase activity which is classically attributed to laccases, and also ferroxidase activity were decreased under Fe deficiency despite the increase in transcript levels of these two *LMCO* genes (Carrió-Seguí et al., 2019b). Previous studies showed that mir408 levels change in opposing directions under Fe and Cu deficiency, respectively (Buhtz et al., 2010; Carrió-Seguí et al., 2019b). On control medium, there was a pronounced increase in *LAC12* transcript levels in the *irt1* mutant compared to the wild-type (Figure 1). This was consistent with a regulation of *LAC12* transcript levels in dependence on plant physiological Fe status, similar to the regulation of a number of other Fe deficiency-responsive genes in roots of the *irt1* mutant (Quintana et al., 2021).

The heterologous expression of the *LAC12* cDNA restored growth of *fet3fet4* yeast cells lacking both the ferroxidase FET3 that is indispensable for high-affinity Fe uptake and FET4-mediated low-affinity Fe^2+^ uptake activity (Figure 2). This result suggested that LAC12 may be able to function as MCO with ferroxidase activity that is required to oxidize Fe(II) to Fe(III) prior to the uptake of Fe(III) into yeast cells by ScFTR1.

To investigate the role of *LAC12* in *A. thaliana*, two *lac12* T-DNA insertion mutants were characterized, in which *LAC12* transcript levels were strongly reduced (Supplementary Figure 3). The two *lac12* mutants were sensitive to Fe deficiency (Figure 3), with reduced shoot and root biomass as well as strongly decreased leaf chlorophyll concentrations (Figure 3). This suggested that *LAC12* function is relevant in the acclimation to Fe deficiency. Under Fe deficiency conditions, *lac12* mutant plants also showed higher Fe concentrations in roots compared to wild-type plants (Figure 4A). We could not detect any differences in shoot Fe concentrations between wild-type and *lac12* mutant seedlings (Figure 4B), but total shoot Fe content was lower in *lac12* mutants compared to WT seedlings under Fe deficiency conditions (Figure 4C). It was previously suggested that under severe Fe deficiency corresponding to Fe limitation, shoot growth decreases rather than shoot Fe concentrations (Baxter et al., 2008). This result may alternatively be in agreement with previously published studies in which Fe deficiency, even before detecting any decrease in Fe concentrations, induces the accumulation of Cu in rosette leaves (Waters et al., 2012; Kastoori Ramamurthy et al., 2018). Perls’ stain detected Fe primarily in the stele of the *lac12* mutant roots grown under Fe deficiency, but not in wild-type roots (Figure 5). Bernal et al. (2012) reported lowered ferroxidase activities in roots of Cu-deficient *spl7* alongside a root-to-shoot Fe translocation defect, and raised the possibility that one or some membrane transport steps in Fe distribution may depend on MCO-mediated ferroxidase activity. If the decrease in ferroxidase activity in Cu-deficient *spl7* was caused solely by impaired LAC12 activity, we would expect Fe to accumulate in the same location in *lac12* and *spl7* roots. Indeed, the localization of Perls’ stain was similar in *lac12* and *spl7* roots, but it was much weaker in *lac12* roots (Figure 5). This may indicate that the decrease in ferroxidase activity in Cu-deficient *spl7* was not caused solely by impaired LAC12 activity. The site of Fe accumulation in roots of *lac12* mutants resembles the site of *LAC12* expression according to the public eFP browser, mainly in the vasculature of the root maturation zone adjacent to the xylem (Supplementary Figure 3A) (Brady et al., 2007; Winter et al., 2007). Lower ferroxidase and phenoloxidase activities observed in Fe-deficient *lac12* roots (Figure 6) were consistent with the possibility that LAC12 is a MCO with ferroxidase activity.

Root-to-shoot Fe translocation may well require more than the single LAC12 protein, which all depend on *SPL7* function under Cu-deficient growth conditions, for example additionally LAC13 or AO, both of them are expressed in the vascular tissues (Supplementary Figure 3A). In the future, generating *lac12 lac13* and *lac12 ao* double mutants will help to clarify their roles. Other candidate proteins that should be examined are LPR1 and LPR2, two MCOs that have a central roles as ferroxidases mediating root phosphate deficiency responses and are expressed, as *LAC12* and *LAC13*, in the vasculature of the root maturation zone adjacent to the xylem (Supplementary Figure 3A) (Müller et al., 2015).

**FIGURE 8:**
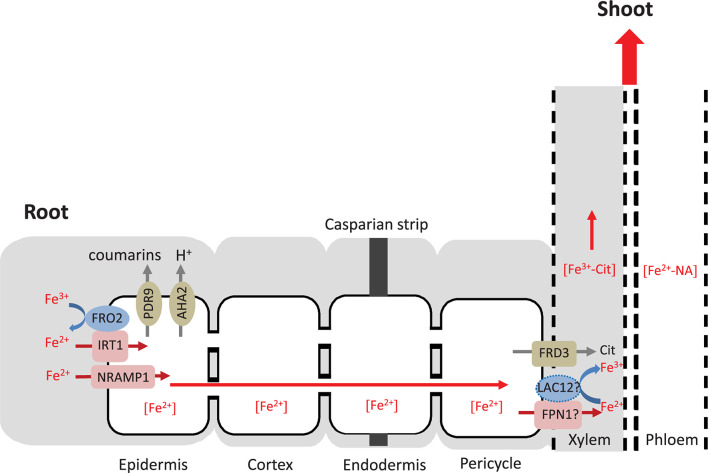
Hypothesis of the role of LAC12 in root Fe homeostasis. The Fe uptake machinery in the root epidermis includes four membrane proteins: PDR9 to exude Fe(III)-binding coumarins, AHA2 to acidify the rhizosphere, FRO2 to reduce Fe(III) to Fe(II) and IRT1 to transport Fe(II) across the plasma membrane into the cytosol (Connorton et al., 2017; Kroh and Pilon, 2020). NRAMP1 contributes to Fe(II) transport as a low-affinity uptake system (Cailliatte et al., 2010). Free or chelated extracellular Fe disseminates or precipitates within the apoplast, and is arrested by the casparian strip that prevents its entry into the stele. Once Fe(II) enters the symplastic pathway of the root epidermal cells, it is probably chelated to a low-molecular-mass ligand molecule, potentially nicotianamine (NA), and can move from cell to cell through plasmodesmata. IRON-REGULATED PROTEIN 1 (also known as ferroportin FPN1) may export Fe(II) from the cytosol of xylem parenchyma cells (not shown) into the xylem vessels (Morrissey et al., 2009). FERRIC REDUCTASE DEFECTIVE 3 (FRD3) drives the efflux of citrate into the xylem, which is critical for root-to-shoot Fe partitioning in the xylem and the leaf apoplast (Green and Rogers, 2004). The primary specie contained in the xylem is an Fe(III)-citrate complex, and consequently it appears that Fe(II) must be oxidized to Fe(III) (Roschzttardtz et al., 2011). According to our hypothesis, LAC12 may be the protein carrying out this function prior to Fe(III) chelation by citrate. Subsequently, the Fe(III)-citrate complex moves upward in the xylem with mass flow. In order to reach sink organs, Fe must be taken up, moved across cells and loaded into the phloem, where it can move as an Fe(II)-NA chelate. According to FACS-transcriptomics results shown in Supplementary Figure 3, LAC12 probably locates in the procambium and not in the pericycle. The dashed line shows the hypothetical localization of LAC12. The apoplast is marked in gray, the symplast in white.

The longitudinal zone of *LAC12* expression corresponds to the zone where roots take up Fe from the soil solution. Consequently, the expression pattern of *LAC12* in roots is generally in accordance with a speculative role of LAC12 in the movement of Fe toward or into the xylem. In analogy with MCO proteins of other organisms which have ferroxidase functions, LAC12 could contribute to Fe oxidation either preceding transmembrane Fe^3+^ uptake into single cells or directly following Fe^2+^ export from xylem parenchyma cells into xylem vessels, for example. The latter function is more likely, given that no ScFTR1/ScFTR5-like genes are present in the Arabidopsis genome (Figure 8). In this respect, transcript levels of *IRON REGULATED 1*/*FERROPORTIN 1* and *2* (*IREG1*/*FPN1, IREG2/FPN2*) were both increased in Fe-deficient WT roots compared to controls (Figure 7), and the expression of *FPN1* was downregulated in Fe-deficient roots of *lac12* mutants. *FPN1* encodes a plasma membrane-localized cellular metal exporter and is expressed in the root stele. Our observation might reflect an interaction of LAC12 and FPN1 protein function at this site, as hypothesized in our working model Figure 8). *FPN2* encodes a vacuolar Fe transporter and is expressed in the root epidermis. *FPN2* transcript levels are upregulated under Fe-deficient conditions (Morrissey et al., 2009). Recently, FPN3, another member of this Fe exporter family, has been characterized. FPN3 is mainly localized in mitochondria and chloroplasts, and its expression increases under Fe deficiency only in roots (Kim et al., 2021).

A preliminary characterization of the *ASCORBATE OXIDASE AO* gene, which is also a candidate ferroxidase (see Supplementary Figure 1), suggested that *ao* mutant seedlings may also be sensitive to Fe deficiency (Supplementary Figure 4). AO is member of the cupredoxin superfamily that has recently been functionally characterized. *AO*, together with three other genes (*BBE22*, *GPX7* and *GSTU4*), was proposed to act in a complex network of ROS-related genes mediating the response of Arabidopsis to the spider mite *Tetranychus urticae* (Santamaría et al., 2018).

In all genotypes grown under Fe-deficient conditions we observed higher root and shoot Cu concentrations than upon cultivation under Fe-sufficient conditions (Supplementary Figure 6). Previous studies reported that Fe deficiency, even before detecting any decrease in Fe concentrations, induces the accumulation of Cu in rosette leaves (Waters et al., 2012; Kastoori Ramamurthy et al., 2018). Under Fe deficiency, shoot responses require interactions with Cu accumulation that mediate the replacement of Fe-containing enzymes by Cu-containing enzymes such as SOD (Waters et al., 2012). Root Cu accumulation under Fe deficiency can be explained by Cu uptake *via* enhanced expression of genes encoding ZRT/IRT-LIKE PROTEIN2 (ZIP2) and COPT2 membrane transporters, which can mediate the cellular uptake of Cu(II) or Cu(I), respectively (Colangelo and Guerinot, 2004; Perea-García et al., 2013).

After a careful analysis of the different studies regarding the cross-talk between Fe and Cu homeostasis in plants, we must admit that the relationship among them is very complex. On the one hand, our previous publication showed that Cu deficiency drives Fe accumulation in the root of wild-type and *spl7* mutant (Bernal et al., 2012). Another laboratory reported that Cu-deficient *spl7* mutant leaves accumulate more Fe, and that lowering Fe supply rescues the growth phenotype of *spl7* mutants (Kastoori Ramamurthy et al., 2018). Additionally, *ysl3* mutants of *B. distachyon* accumulated more Fe in roots and leaves under Cu-deficient conditions (Sheng et al., 2021). One main difference that could explain the different results obtained by different research groups is the Fe chelator used in the Hoagland’s growth medium: Fe(III)-EDDHA (Kastoori Ramamurthy et al., 2018; Sheng et al., 2021) *versus* FeHBED (Bernal et al., 2012). Our laboratory uses HBED (*N*,*N*′-di(2-hydroxybenzyl)ethylenediamine- *N*,*N*′-diacetic acid) for Fe chelation in plant growth media. This chelator shows only a limited affinity for Cu and remains chelated to Fe(III) over a wide pH range. However, EDDHA has a higher affinity for Cu than for Fe(III) and when added to the growth media can therefore restrict the bioavailability of Cu, among other micronutrients (i.e., Zn), and result in an effective deficiency of these metals. Lowering Fe(III)EDDHA means lowering also EDDHA and thus decreasing Cu chelation. Taking this in consideration, the rescue of the *spl7* phenotype by low Fe supply (Kastoori Ramamurthy et al., 2018) may be correlated with the decrease of the concentration of a Cu chelator and not with lowering Fe concentration itself. In fact, we believe that the strong phenotype of the *spl7* mutant grown under 0.1 μM Cu and 25 μM Fe (Kastoori Ramamurthy et al., 2018) is caused by both, the low Cu concentrations and the effect that Fe(III)-EDDHA has on the bioavailability of Cu.

## Conclusion

Our initial characterization of *LACCASE12* (*LAC12*), a member of the laccase multicopper oxidase (LMCO) protein family, provides evidence for a role in the performance of Arabidopsis on Fe-deficient substrates and initial support for a ferroxidase function of the encoded protein. Alterations in root Fe concentrations and total shoot Fe of *lac12* mutants are consistent with a possible function of LAC12 in root-to-shoot Fe partitioning and a possible role in enabling the cellular export of Fe. More work will clearly be needed to understand the role of MCO proteins LAC12 and the AO in Fe homeostasis. Unraveling the interactions between Cu and Fe homeostasis may lead to the development of novel strategies for combating nutritional Fe deficiencies in crops and for advancing biofortification approaches.

## Data Availability Statement

The original contributions presented in the study are included in the article/Supplementary Material, further inquiries can be directed to the corresponding authors.

## Author Contributions

MB and UK conceived the project, designed research, and analyzed data. MB performed experiments and wrote the manuscript. UK commented on and edited the manuscript. Both authors contributed to the article and approved the submitted version.

## Conflict of Interest

The authors declare that the research was conducted in the absence of any commercial or financial relationships that could be construed as a potential conflict of interest.

## Publisher’s Note

All claims expressed in this article are solely those of the authors and do not necessarily represent those of their affiliated organizations, or those of the publisher, the editors and the reviewers. Any product that may be evaluated in this article, or claim that may be made by its manufacturer, is not guaranteed or endorsed by the publisher.

## Footnotes

1
http://www.who.int/nutrition/topics/ida/en/
