# Corrigendum: Involvement of Arabidopsis Multi-Copper Oxidase-Encoding *LACCASE12* in Root-to-Shoot Iron Partitioning: A Novel Example of Copper-Iron Crosstalk

**DOI:** 10.3389/fpls.2021.813380

**Published:** 2021-12-03

**Authors:** María Bernal, Ute Krämer

**Affiliations:** ^1^Department of Molecular Genetics and Physiology of Plants, Faculty of Biology and Biotechnology, Ruhr University Bochum, Bochum, Germany; ^2^Department of Plant Nutrition, Estación Experimental de Aula Dei-CSIC, Zaragoza, Spain

**Keywords:** copper, iron, multicopper oxidase, homeostasis, deficiency

In the original article, there was a mistake in Figure 2B as published. The panel B showed the positive control of panel A and it was accidentally taken from a different experiment when the figure was prepared. The corrected Figure 2 with the correct positive control plate appears below.

**Figure 2:**
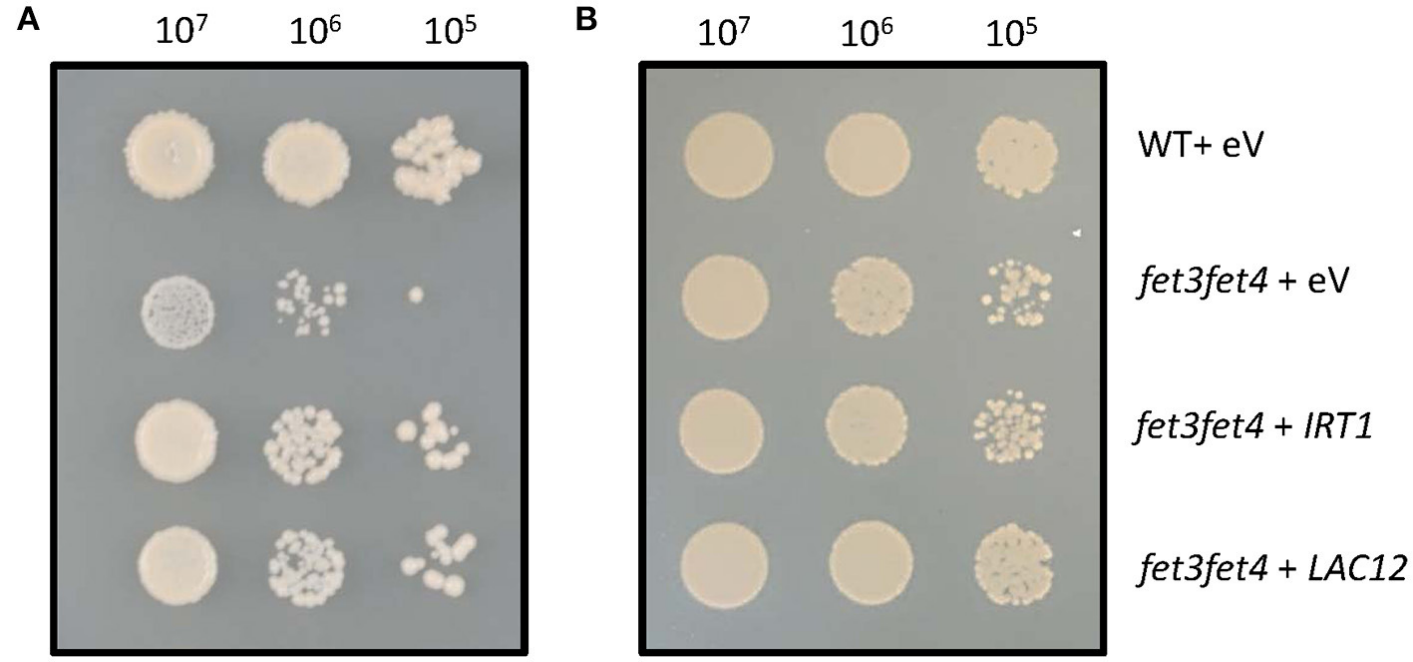
Heterologous expression of the *AtLAC12* cDNA complements a Fe uptake-defective *fet3fet4* mutant of *Saccharomyces cerevisiae*. Wild-type and the Fe uptake-defective *fet3fet4* mutant of *Saccharomyces cerevisiae* transformed with the empty vector pFL61 Gateway (eV) or expressing *IRT1* or *LAC12* cDNAs of *A. thaliana*, respectively. Aliquots of 10 μL of 10-fold serial dilutions (starting from OD_600_ = 0.3, *ca*. 10^7^ cells ml^−1^) were spotted on **(A)** SD-URA medium (pH 5.7) and **(B)** SD-URA medium (pH 5.7) supplemented with 0.5 mM FeSO_4_. Images are representative of three independent transformant colonies from each of two independent experiments.

The authors apologize for this error and state that this does not change the scientific conclusions of the article in any way. The original article has been updated.

## Publisher's Note

All claims expressed in this article are solely those of the authors and do not necessarily represent those of their affiliated organizations, or those of the publisher, the editors and the reviewers. Any product that may be evaluated in this article, or claim that may be made by its manufacturer, is not guaranteed or endorsed by the publisher.

