# Supplementary material for: Involvement of Arabidopsis Multi-Copper Oxidase-Encoding LACCASE12 in Root-to-Shoot Iron Partitioning: A Novel Example of Copper-Iron Crosstalk
Source: Front Plant Sci. 2021 Oct 11;12:688318. doi: 10.3389/fpls.2021.688318 (PMC8544784; doi:10.3389/fpls.2021.688318)
Supplement: Supplementary file 1 [file Data_Sheet_1.zip › Supplementary Figure 1.pdf]

```
ScFET3  1    MTN----------------ALLSIAVL-----------------------------------------LF  13
LAC1    1    MEN------LGFLIIST--FLLLFTTL------------------------------------------L  20
LAC2    1    MVT------WVLNYLLV--AFLFAISY-----------------------------------------NI  21
LAC3    1    MES---------FRRFS--LLSFIALLA----------------------------------------YF  19
LAC4    1    MGS-------HMVWFLF--LVSFFSV-------------------------------------------F  18
LAC5    1    MDV---TKSLLCFISFV--AFLLFSSV-------------------------------------------  22
LAC6    1    MTS----SAVPSLFRLS--FLLFTLQV-----------------------------------------MN  23
LAC7    1    MEG-VRVP-----------IACALILL-----------------------------------------AI  17
LAC8    1    MPR--------LHHYLS--NQAFLVLL-----------------------------------------LF  19
LAC9    1    MPR--------VHHSLS--NQAFLVLL-----------------------------------------LF  19
LAC10   1    M-----------VFPIR--ILVLFALL-----------------------------------------AF  16
LAC11   1    MKMG----------FLF--LFCYLLAF-----------------------------------------LG  17
LAC12   1    MTT---------VHTFS--ILLFFCSL-----------------------------------------FS  18
LAC13   1    MEQ---------LRPF------FLLLAI----------------------------------------FV  15
LAC14   1    MEFKLNIPNTIIKTLQT--IVFFLFVL-----------------------------------------LA  27
LAC15   1    MSH----------------SFFNLFLI-----------------------------------------SL  13
LAC16   1    M---------------------------------------------------------------------  1
LAC17   1    MAL-----------QLL--LAVFSCVL-----------------------------------------LL  16
AO      1    MMRPKRSSDTVHVFNLM--VLCFIALF-----------------------------------------FS  27
LPR1    1    MES------LLCRRRIKR-VMVLIIALTWLRSTCG---ELEDQLFEVGKLKMFVDDLPDMPRLYGFNSVH  60
LPR2    1    MEP--------SRRRMTRDMLLLIVTMAWLVTGDEGGIKQEERLFNLGKLEMFVDKLPHIPTLHGYHFVN  62

ScFET3  14   SMLSLAQAETHTFNWTTGWDY----RNVDGL-KSRPVITCNGQFPWPDITVNKGDRVQIYLTNGMNNTN-  77
LAC1    21   PYSSASTTRRFHFNVEW--------KKVTRLCHTKQLLTVNGQYPGPTVAVHEGDIVEIKVTNR-IAHN-  80
LAC2    22   DAASAGITRHYQFDIQL--------KNITRLCKTKTIVTVNGKFPGPRVTAREGDNLQIKVVNH-VSNN-  81
LAC3    20   AFLASAEHHVHQFVITP--------TPVKRLCRTHQSITVNGQYPGPTLVVRNGDSLAITVINR-ARYN-  79
LAC4    19   PAPSESMVRHYKFNVVM--------KNVTRLCSSKPTVTVNGRYPGPTIYAREDDTLLIKVVNH-VKYN-  78
LAC5    22   --AEANKAHHHEFIIQA--------TKVKRLCETHNSITVNGMFPGPMLVVNNGDTLVVKVINR-ARYN-  80
LAC6    24   IGRIGAATRFYQFKVQT--------IRLTRLCQTNEIVTVNKKFPGPAISAQEDDRIVIKVINM-TPYN-  83
LAC7    18   SSITSASIVEHTFNVQN--------LTVSRLCKRQVITVVNGSLPGPTIRVKEGDSLVIHVLNH-SPHN-  77
LAC8    20   SSIASAAVVEHVLHIQD--------VVVKPLCKEQIIPAANGSLPGPTINVREGDTLVVNVINN-STYN-  79
LAC9    20   SSIASAAIVEHVLHVKD--------VVVTPLCKEQMIPIVNGSLPGPTINVREGDTLVVHVINK-STYN-  79
LAC10   17   PACVHGAIRKYTFNVVT--------KQVTRICSTKQIVTVNGKFPGPTIYANEDDTILVNVVNN-VKYN-  76
LAC11   18   YSPVDAAVKKYQFDVQV--------KNISRICNAKPIVTVNGMFPGPTVYAREGDRVIINVTNH-VQYN-  77
LAC12   19   ASLIIAKVQHHDFVIQE--------TPVKRLCKTRNAITVNGMFPGPTLEVNNGDTLEVKVHNR-ARYN-  78
LAC13   16   ASLVNAEVHFHEFVIQE--------TPVKRLCRVHNSITVNGQFPGPTLEVRNGDSLVITAINK-ARYN-  75
LAC14   28   FQIAEAEIHHHTFKIKS--------KAYTRLCNTNKILTVNGEFPGPTLKAYRGDKLIVNVINN-ANYN-  87
LAC15   14   FLYNNCIAHHYTFTVRE--------VPYTKLCSTKAILTVNSQFPGPIIKVHKGDTIYVNVQNR-ASEN-  73
LAC16   1    -------------------------TNTTKLCSSKPIVTVNGQFPGPTIVAREGDTILIKVVNH-VKYN-  44
LAC17   17   PQPAFGITRHYTLEIKM--------QNVTRLCHTKSLVSVNGQFPGPKLIAREGDQVLIKVVNQ-VPNN-  76
AO      28   SVLCQGKIRRFKWEVKY--------EFKSPDCFEKLVITINGKFPGPTIKAQQGDTIVVELKNSFMTEN-  88
LPR1    61   GIIKPASLQIGMFSTKWKFHRDLPATPVFAYGTSRSKATV----PGPTIETVYGVDTYVTWRNH-LPKSH  125
LPR2    63   GFLKPKSLHIGMFFKKWKFHRDLPATPVFAYGTSKRSATV----PGPTIEAVYGVDTYVTWRNH-LPLHH  127

ScFET3  77   -------------------TSMHFHGLFQNGTASMDGVPFLTQCPIAPGSTM---LYNFTVDYNVGTYWY  125
LAC1    80   -------------------TTIHWHGLRQYRTGWADGPAYITQCPIRSKQSY---TYRFKVEDQRGTLLW  128
LAC2    81   -------------------ISIHWHGIRQLRSGWADGPSYVTQCPIRMGQSY---VYNFTVTGQRGTLWW  129
LAC3    79   -------------------ISIHWHGIRQLRNPWADGPEYITQCPIRPGQTY---TYRFKIEDQEGTLWW  127
LAC4    78   -------------------VSIHWHGVRQVRTGWADGPAYITQCPIQPGQVY---TYNYTLTGQRGTLWW  126
LAC5    80   -------------------ITIHWHGVRQMRTGWADGPEFVTQCPIRPGSSY---TYRFTIQGQEGTLWW  128
LAC6    83   -------------------TTIHWHGIKQKRSCWYDGPSYITQCPIQSGQSF---TYNFKVAQQKGTFLW  131
LAC7    77   -------------------ITIHWHGIFHKLTVWADGPSMITQCPIQPGQRY---AYRFNITGQEGTLWW  125
LAC8    79   -------------------VTIHWHGVFQLKSVWMDGANMITQCPIQPGYNF---TYQFDITGQEGTLLW  127
LAC9    79   -------------------VTIHWHGVFQLKSVWMDGANMITQCPIQPSNNF---TYQFDITGQEGTLLW  127
LAC10   76   -------------------VSIHWHGIRQLRTGWADGPAYITQCPIKPGHSY---VYNFTVTGQRGTLWW  124
LAC11   77   -------------------MSIHWHGLKQYRNGWADGPAYITQCPIQTGQSY---LYDFNVTGQRGTLWW  125
LAC12   78   -------------------ITIHWHGVRQIRTGWADGPEFVTQCPIRPGKSY---TYRFTIQGQEGTLWW  126
LAC13   75   -------------------ISLHWHGIRQMRNPWADGPEYITQCPIRPGGSY---TYRFTMEDQEGTLWW  123
LAC14   87   -------------------ITLHWHGARQIRNPWSDGPEYVTQCPIRPGESY---VYRIDLKVEEGTIWW  135
LAC15   73   -------------------ITMHWHGVEQPRNPWSDGPEYITQCPIRPGSDF---LYKVIFSIEDTTVWW  121
LAC16   44   -------------------VSIHW-------TGWADGPAYITQCPIQPGQNY---LHNFTLTGQRGTLWW  85
LAC17   76   -------------------ISLHWHGIRQLRSGWADGPAYITQCPIQTGQSY---VYNYTIVGQRGTLWY  124
AO      88   -------------------VAVHWHGIRQIGTPWFDGVEGVTQCPILPGEVF---IYQFVVD-RPGTYMY  135
LPR1    126  ILPWDPTISPATPKHGGIPTVVHLHGGIHEPTSDGNADAWFTAGFRETGPKWTKTTLHYENKQQPGNMWY  195
LPR2    128  ILPWDPTISPAIPKHGGIPTVVHLHGGIHEPTSDGNADSWFTAGFKETGSKWTKKTTHYVNKQQPGNMWY  197
```

```
ScFET3   126 HSHTDGQ-YED----GMKGLFIIKDDSFP-YDYD--------EELSLSLSEWYHDL-VTDLTKSFMSVYN 180
LAC1     129 HAHHSWQ-RAS----VYGAFIIYPR--QP-YPFS-GSHIQ--SEIPIILGEWWNDD-VDNVEKAMMK--- 183
LAC2     130 HAHIQWM-RAT----VYGPLIILPKLHQP-YPFP-KPY----KQVPILFGEWFNAD-PQAVVQQALQ--- 184
LAC3     128 HAHSRWL-RAT----VYGALIIYPRLGSP-YPFS-MPK----RDIPILLGEWWDRN-PMDVLKQAQF--- 182
LAC4     127 HAHILWL-RAT----VYGALVILPKRGVP-YPFP-KPD----NEKVIVLGEWWKSD-TENIINEALK--- 181
LAC5     129 HAHSSWL-RAT----VYGSLLVFPPAGSS-YPFT-KPH----RNVPLLLGEWWDAN-PVDVLRESIR--- 183
LAC6     132 HAHFSWL-RAT----VYGPLIVYPKASVP-YPFK-KPF----NEHTILLGEYWLKN-VVELEQHVLE--- 186
LAC7     126 HAHASFL-RAT----VYGALVIRPKSGHS-YPFP-KPH----KEVPILFGEWWNTD-VVALEEAAIA--- 180
LAC8     128 HAHVVNL-RAT----LHGALVIRPRSGRP-YPFP-KPY----KEVPIVFQQWWDTD-VRLLQ-------- 177
LAC9     128 HAHVVNL-RAT----IHGALIIRPRSGRP-YPFP-KPY----KEVPLIFQQWWDTD-VRLLE-------- 177
LAC10    125 HAHVLWL-RAT----VHGAIVILPKLGLP-YPFP-KPH----REEVIILGEWWKSD-TETVVNEALK--- 179
LAC11    126 HAHILWL-RAT----VYGAIVILPAPGKP-YPFP-QPY----QESNIILGEWWNKD-VETAVNQANQ--- 180
LAC12    127 HAHSSWL-RAT----VYGALIIHPTPGSS-FPFP-KPD----RQTALMLGEWWNAN-PVDVINQATR--- 181
LAC13    124 HAHSRWL-RAT----VYGALIIRPPLSSPHYPFPVIPK----REITLLLGEWWDRN-PMDVLNLAQF--- 180
LAC14    136 HAHSQWA-RAT----VHGAFIVYPKRGSS-YPFP-KPH----REIPLILGEWWKKENIMHIPGKANK--- 191
LAC15    122 HAHSSWT-RAT----VHGLIFVYPRPPQI-LPFP-KAD----HEVPIILGEWWKRD-VREVVEEFVR--- 176
LAC16    86  HAHILWL-RAT----VHGAIVILPKLGVP-YPFP-KPY----KEKTIVLSEWWKSD-VEELINEASR--- 140
LAC17    125 HAHISWL-RST----VYGPLIILPKRGVP-YPFA-KPH----KEVPMIFGEWFNAD-TEAIIRQATQ--- 179
AO       136 HSHYGMQ-RES---GLIGMIQVSPPATEP-EPFT-YDY-----DRNFLLTDWYHKS----MSEKATG--- 187
LPR1     196 HDHAMGLTRVNLLAGLVGAYILRHHAVES--PFQ-LPTGDEFDRPLIIFDRSFRKD-GSIYMNA------ 255
LPR2     198 HDHAAGLTRVNLLAGLLGSYILRHSSVES--PLR-LPTGREFDRPLVIFDRSFRKD-GSIYMNA------ 257

ScFET3   181 PTGAEP----------IPQNLIVNNTMN-----------------------------LTWEVQPDTTYLL 211
LAC1     183 -TGAGAK---------VSDAYTLNGLPGPLYPCSTKDT-------------------FTATVDAGKTYIL 224
LAC2     184 -TGAGPN---------ASDAHTFNGLPGPLYNCSTKDT-------------------YKLMVKPGKTYLL 225
LAC3     182 -TGAAAN---------VSDAYTINGQPGDLYRCSRAGT-------------------IRFPIFPGETVQL 223
LAC4     181 -SGLAPN---------VSDSHMINGHPGPVRNCPSQG--------------------YKLSVENGKTYLL 221
LAC5     183 -TGGAPN---------NSDAYTINGQPGDLYKCSSQDT-------------------TVVPINVGETILL 224
LAC6     186 -SGGPPP---------PADAFTINGQPGPNYNCSSKDV-------------------YEIQIVPRKIYLL 227
LAC7     180 -TGVPPN---------NSDAYTINGRPGNLYPCSKDRM-------------------FSLNVVKGKRYLL 221
LAC8     177 -LRPAP----------VSDAYLINGLAGDSYPCSENRM-------------------FNLKVVQGKTYLL 217
LAC9     177 -LRPAP----------VSDAYLINGLAGDSYPCSKNRM-------------------FNLKVVQGKTYLL 217
LAC10    179 -SGLAPN---------VSDAHVINGHPGFVPNCPSQGN-------------------FKLAVESGKTYML 220
LAC11    180 -LGAPPP---------MSDAHTINGKPGPLFPCSEKHT-------------------FVIEAEAGKTYLL 221
LAC12    181 -TGAAPN---------ISDAYTINGQPGDLYNCSTKET-------------------VVVPINSGETSLL 222
LAC13    180 -TGAAPN---------ISDAFTINGQPGDLYRCSSQET-------------------LRFLVGSGEIVLL 221
LAC14    191 -TGGEPA---------ISDSYTINGQPGYLYPCSKPET-------------------FKITVVRGRRYLL 232
LAC15    176 -TGGAPN---------VSDALTINGHPGFLYPCSKSDT-------------------FHLTVEKGKTYRI 217
LAC16    140 -IGTAPS---------ASDAHTINGHSGSISNCPSQSS-------------------YGLPVRAGKTYML 181
LAC17    179 -TGGGPN---------VSDAYTINGLPGPLYNCSAKDT-------------------FRLRVKPGKTYLL 220
AO       187 -LASIPF---KWVG--EPQSLMIQGRG--RFNCSNNLTTPPSLVSGVCNVSNADCSRFILTVIPGKTYRL 249
LPR1     255 -TGNNPSIHPQWQPEYFGDVIIVNGKAWPRLN------------------------------VRRRKYRF 294
LPR2     257 -TGNNPTIHPQWQPEYFGDAIIVNGKAWPRLT------------------------------VRRRKYRF 296

ScFET3   212 RIVNVGGFVSQ-YFWIEDHEMTVVEIDGITT-EKNVTDMLYITVAQRYTVLVHTKND-TDKNFAIMQK-- 276
LAC1     225 RIINAALNNEL-FVAVANHTLTVVEVDAVYT-KPVHTKAIMIAPGQTTTLLLRADQL-SGGEFLIAAT-- 289
LAC2     226 RLINAALNDEL-FFTIANHTLTVVEADACYV-KPFQTNIVLLGPGQTTNVLLKTKPIYPNATFYMLAR-- 291
LAC3     224 RVINAGMNQEL-FFSVANHQFTVVETDSAYT-KPFTTNVIMIGPGQTTNVLLTANQR-PG-RYYMAAR-- 287
LAC4     222 RLVNAALNEEL-FFKVAGHIFTVVEVDAVYV-KPFKTDTVLIAPGQTTNVLLTASKS-AG-KYLVTAS-- 285
LAC5     225 RVINSALNQPL-FFTVANHKLTVVGADASYL-KPFTTNVIVLGPGQTTDVLITGDQP-PN-RYYMAAR-- 288
LAC6     228 RLINAGINMET-FFTIANHRLTIVEVDGEYT-KPYTTERVMLVPGQTMNILVTADQT-VG-RYSMAMG-- 291
LAC7     222 RIINAAMNIQL-FFKIANHRLTVVAADAVYT-APYVTDVIVIAPGQTIDALLFADQS-VDTSYYMAAH-- 286
LAC8     218 RIVNAALNTHL-FFKIANHNVTVVAVDAVYS-TPYLTDVMILTPGQTVDALLTADQA-IG-KYYMATL-- 281
LAC9     218 RIINAALNTHL-FFKIANHNVTVVAVDAVYT-TPYLTDVMILTPGQTIDAILTADQP-IG-TYYMAII-- 281
LAC10    221 RLINAALNEEL-FFKIAGHRFTVVEVDAVYV-KPFNTDTILIAPGQTTTALVSAARP-SG-QYLIAAA-- 284
LAC11    222 RIINAALNDEL-FFGIAGHNMTVVEIDAVYT-KPFTTKAILLGPGQTTNVLVKTDRS-PN-RYFMAAS-- 285
LAC12    223 RVINAALNQPL-FFTVANHKLTVVGADASYL-KPFTTKVLMLGPGQTTDVLLTADQP-PK-RYYIAAR-- 286
LAC13    222 RVINSALNQEL-FFGVANHKLTVVAADASYT-KPFSTNVIMLGPGQTTDVLLTADQP-PA-HYYMAAH-- 285
LAC14    233 RIINAVMDEEL-FFAIANHTLTVVAKDGFYL-KHFKSDYLMITPGQSMDVLLHANQR-PN-HYFVAAR-- 296
LAC15    218 RMVNAAMNLPL-FFAIANHSLTVVSADGHYI-KPIKATYITISPGETLDMLLHADQD-PERTYYMAAR-- 282
LAC16    182 RIINAALNEEL-FFKIAGHVLTVVEVDAVYT-KPYKTDTVFIAPGQTTNVLLTANAN-AGSNYMVAAT-- 246
LAC17    221 RLINAALNDEL-FFSIANHTVTVVEADAIYV-KPFETETILIAPGQTTNVLLKTKSSYPSASFFMTAR-- 286
AO       250 RIGSLTALSAL-SFQIEGHNLTVVEADGHYV-EPFTVKNLFVYSGETYSVLLKADQN-PRRNYWITSSIV 316
LPR1     295 RIINASNARFFKFFFSNGLDFIVVGSDSAYLSKPVMTKSILLSPSEIVDVVVDFYKS-PSRTVVLANDAP 363
LPR2     297 RITNASNARFFRFFFSNGLDFIVVGSDSAYLAKPVSTKSVLLAPSEIVDVLVDFSKS-TSKTAILANNAP 365
```

```
ScFET3   276 ------------FDDTMLDVIPSDLQLNATSY-------------MVYNK-TAALPTQNYVDSIDNFLDD 320
LAC1     289 -PYVTS-VFP--FNNST---TVGFIRYTGKTKPENSVNTRRRRRLTAMST-VVALPNMLDTKFATKFSDS 351
LAC2     291 -PYFTG-QGT--IDNTT---VAGILQYQHHTKS----------SKNLSII-KPSLPPINSTSYAANFTKM 343
LAC3     287 -AYNSA-NAP--FDNTT---TTAILQYVNAPTRRGRG------RGQIAPV-FPVLPGFNDTATATAFTNR 343
LAC4     285 -PFMDA-PIA--VDNVT---ATATVHYSGTLS-------------SSPTI-LTLPPPQNATSIANNFTNS 334
LAC5     288 -AYQSAQNAP--FGNTT---TTAILQYKSAPCCGVGGGSGTKKGNSFKPI-MPILPAYNDTNTVTRFSQS 351
LAC6     291 -PYESAKNVK--FQNTS---AIANFQYIGALP-------------NNVTV-PAKLPIFNDNIAVKTVMDG 341
LAC7     286 -PYASAPAVP--FPNTT---TRGVIHYGGASK-----------TGRSKPVLMPKLPSFFDTLTAYRFYSN 339
LAC8     281 -PYISAIGIP-TPDIKP---TRGLIVYQGATS-------------SSSPA-EPLMPVPNDMSTAHRFTSN 332
LAC9     281 -PYFSAIGVPASPDTKP---TRGLIVYEGATS-------------SSSPT-KPWMPPANDIPTAHRFSSN 333
LAC10    284 -PFQDSAVVA--VDNRT---ATATVHYSGTLS-------------ATPTK-TTSPPPQNATSVANTFVNS 334
LAC11    285 -PFMDA-PVS--VDNKT---VTAILQYKGVPN-------------TVLPI-LPKLPLPNDTSFALDYNGK 334
LAC12    286 -AYQSAQNAP--FDNTT---TTAILQYKKTTT-------------TSKPI-MPVLPAFNDTNTVTSFSRK 336
LAC13    285 -AYNSA-NAA--FDNTT---TTAILKYKDASCVTLQA------KSQARAI-PAQLPGFNDTATAAAFTAQ 341
LAC14    296 -AYSSAFGAG--FDKTT---TTAILQYKGDTL------------NRIKPI-LPYLPPYNRTEASTRFTNQ 347
LAC15    282 -AYQSG-NID--FNNST---TIGILSYTSSCKAK---------TSSFSGY-YPTLPFYNDTSAAFGFFTK 335
LAC16    246 -TFTDA-HIP--YDNVT---ATATLHYIGHTSTV---------STSKKTV-LASLPPQNATWVATKFTRS 299
LAC17    286 -PYVTG-QGT--FDNST---VAGILEYEPPKQTKGAHSRTS--IKNLQLF-KPILPALNDTNFATKFSNK 346
AO       316 -------SRP--ATTPP---ATAVLNYYPNHPRR---------RPPTSES-SNIVPEWNDTR--SRLAQS 362
LPR1     364 YPYPSGDPVN--EENGK--VMKFIINNESEDD-----------TCTIPKKLINYPNADVSNAVLTRYISM 418
LPR2     366 YPYPSGDPVT--EENSK--VMKFIINYKSEVD-----------TSIIPKKLIEYPPAHVSTSTRTRYIAM 420

ScFET3   321 FYLQPY----EKEAIYGEPDHVITVDVVMDNLK-------------NGVN-YAFFNNITYTAPKV-PTLMT 372
LAC1     352 IKSLGSAKYPCKVPTKIDKRVITTISLNLQDCP---LNQTCDGY-AGKRFFASMNNISFVRPPI-SILES 416
LAC2     344 FRSLASSTFPANVPKVVDKQYFFAIGLGTNPCP---KNQTCQGPTNTTKFAASINNVSFILPNKTSLLQS 410
LAC3     344 LRYWKR----APVPQQVDENLFFTVGLGLINCANP-NSPRCQGP-NGTRFAASMNNMSFVLPRSNSVMQA 407
LAC4     335 LRSLNSKKYPALVPTTIDHHLFFTVGLGLNACP------TCKAG-NGSRVVASINNVTFIMPKT-ALLPA 396
LAC5     352 FRSLRR----AEVPTEIDENLFVTIGLGLNNCPKNFRSRRCQGP-NGTRFTASMNNVSFALPSNYSLLQA 416
LAC6     342 LRSLNA----VDVPRNIDAHLFITIGLNVNKCNSENPNNKCQGP-RKGRLAASMNNISFIEPKV-SILEA 405
LAC7     340 LTALVNGPHWVPVPRYVDEEMLVTIGLGLEACA---DNTTC-------PKFSASMSNHSFVLPKKLSILEA 400
LAC8     333 ITSLVGGPHWTPVPRHVDEKMFITMGLGLDPCP---AGTKCIGP-LGQRYAGSLNNRTFMIPERISMQEA 398
LAC9     334 ITSLVGGPHWTPVPRHVDEKMFITMGLGLDPCP---SNAKCVGP-LDQRLAGSLNNRTFMIPERISMQEA 399
LAC10    335 LRSLNSKTYPANVPITVDHDLLFTVGLGINRCH------SCKAG-NFSRVVAAINNITFKMPKT-ALLQA 396
LAC11    335 LKSLNTPNFPALVPLKVDRRLFYTIGLGINACP------TCV---NGTNLAASINNITFIMPKT-ALLKA 394
LAC12    337 FKSLRN----VVVPKTIDDNLFFTIGLGLDNCPKKFPKSRCQGL-NGTRFTASMNNVSFVLPSNFSLLQA 401
LAC13    342 MKSPSK----VKVPLEIDENLFFTVGLGLFNCPTP-NTQRCQGP-NGTRFTASINNVSFVFPKQNSIMQA 405
LAC14    348 FRSQRP----VNVPVKINTRLLYAISVNLMNCS---DDRPCTGP-FGKRFSSSINNISFVNPSV-DILRA 408
LAC15    336 IKCLFS----GQVPVQISRRIITTVSINLRMCP---QN-SCEGP-NGSRLAASMNNISFVTPSHVDILKA 396
LAC16    300 LRSLNSLEYPARVPTTVEHSLFFTVGLGANPCQ------SCN---NGVRLVAGINNVTFTMPKT-ALLQA 359
LAC17    347 LRSLNSKNFPANVPLNVDRKFFFTVGLGTNPCNHK-NNQTCQGPTNTTMFAASISNISFTMPTK-ALLQS 414
AO       363 LAIKARRGFIHALPENSDKVIVL---LNTQNEV-------------NGYR-RWSVNNVSYHHPKT-PYLIA 415
LPR1     419 YEYVSN----SDEPTHL------------------------------------LVNGLPYEAPVT------ 443
LPR2     421 FEYVSS----IDEPTHL------------------------------------YINGLPYNAPVT------ 445

ScFET3   373 VLSSGD-------------------------QA-NNSEIYGSNTHTFILEKDEIVEIVLNNQDT----- 410
LAC1     417 YYKKQSKGVFSLDFPEKPPNRFDFTG--VDPV---SENMNTEFGTKLFEVEFGSRLEIVFQGTSFLNIE- 480
LAC2     411 YFVGKSKNVFMTDFPTAPIIPFNYTG--TPP-----NNTMVSRGTKVVVLKYKTTVELVLQGTSILGIE- 472
LAC3     408 YYQGTP-GIFTTDFPPVPPVQFDYTG---NVS---RGLWQPIKGTKAYKLKYKSNVQIVLQDTSIVTPE- 469
LAC4     397 HYFNTS-GVFTTDFPKNPPHVFNYSG--GSV-----TNMATETGTRLYKLPYNATVQLVLQDTGVIAPE- 457
LAC5     417 HHHGIP-GVFTTDFPAKPPVKFDYTG--NNIS---RSLYQPDRGTKLYKLKYGSRVQIVLQDTGIVTPE- 479
LAC6     406 YYKQLE-GYFTLDFPTTPEKAYDFVNGAPNDI---ANDTQAANGTRAIVFEYGSRIQIIFQNTGTLTTE- 470
LAC7     401 VFHDVK-GIFTADFPDQPPVKFDYTN--PNVTQTNPGLLFTQKSTSAKILKFNTTVEVVLQNHALIAAE- 466
LAC8     399 YFYNIS-[illegible]IY[illegible]DD[illegible]NQ[illegible]LK[illegible]KFEQR-TNNDMKMMFPERKTSVKKIRFNSTVEIVLQNTAIISPE- 465
LAC9     400 YFYNIT-GVYTDDFPDQPPLKFDFTKFEQHPTNSDMEMMFPERKTSVKTIRFNSTVEIVLQNTGILTPE- 467
LAC10    397 HYFNLT-GIYTTDFPAKPRRVFDFTG--KPP-----SNLATMKATKLYKLPYNSTVQVVLQDTGNVAPE- 457
LAC11    395 HYSNIS-GVFRTDFPDRPPKAFNYTG--VPLT----ANLGTSTGTRLSRVKFNTTIELVLQDTNLLTVE- 456
LAC12    402 HSNGIP-GVFTTDFPSKPPVKFDYTG--NNIS---RALFQPVKGTKLYKLKYGSRVQVVLQDTNIVTSE- 464
LAC13    406 YYQGTPTGVFTTDFPPTPPVTFDYTG---NVS---RGLWQPTRGTKAYKLKFNSQVQIILQDTSIVTTE- 468
LAC14    409 YYRHIG-GVFQEDFPRNPPTKFNYTG--ENLP------FPTRFGTKVVVLDYNSSVELILQGTTVWASN- 468
LAC15    397 YYYHIK-GVYGTRFPEFPPLIFNFTA--ENQP---LFLETPRLATEVKVIEFGQVVELVIQGTSLVGGG- 459
LAC16    360 HFFNIS-[illegible]DD[illegible]AK[illegible]SNPY[illegible]A--PVKL---GVNAATMKGTKLYRLPYNATVQIVLQNTAMILSD- 422
LAC17    415 HYSGQSHGVYSPKFPWSPIVPFNYTG--TPP-----NNTMVSNGTNLMVLPYNTSVELVMQDTSILGAE- 476
AO       416 LKQNLT-NAFDWRF--TAPENYDSRN--YDIFAK-PLNANATTSDGIYRLRFNSTVDVILQNANTMNANN 479
LPR1     443 --------------------------------------ETPKSGT--------TEVWEVIN----LTED- 462
LPR2     445 --------------------------------------ETPKIGT--------SEVWEVIN----LTED- 464
```

```
ScFET3  410 -GTHPFHLHGHAFQTIQRDRTYDDALGEVPHSFD----PDNHP-AFPEY--PMR--RDTLYVRPQSN--- 467
LAC1    480 --NHPLHVHGHNFFVV----------GRGFGNFD----PEKDP-KRYNLVDPPE--RNTFAVPTGGW--- 528
LAC2    472 --AHPIHLHGFNFYVV----------GQGFGNFN----PARDP-KHYNLVDPVE--RNTINIPSGGW--- 520
LAC3    469 --NHPMHLHGYQFYVV----------GSGFGNFN----PRTDP-ARFNLFDPPE--RNTIGTPPGGW--- 517
LAC4    457 --NHPVHLHGFNFFEV----------GRGLGNFN----STKDP-KNFNLVDPVE--RNTIGVPSGGW--- 505
LAC5    479 --NHPIHLHGYDFYII----------AEGFGNFN----PKKDT-AKFNLEDPPL--RNTVGVPVNGW--- 527
LAC6    470 --NHPIHLHGHSFYVI----------GYGTGNYD------QQT-AKFNLEDPPY--LNTIGVPVGGW--- 516
LAC7    466 --SHPMHLHGFNFHVL----------AQGFGNYD----PSRDR-SKLNLVDPQS--RNTLAVPVGGW--- 514
LAC8    465 --SHPMHLHGFNFYVL----------GYGFGNYD----PIRDA-RKLNLFNPQM--HNTVGVPPGGW--- 513
LAC9    467 --SHPMHLHGFNFYVL----------GYGFGNYD----PIRDA-RKLNLFNPQM--HNTVGVPPGGW--- 515
LAC10   457 --NHPIHLHGFNFFVV----------GLGTGNYN----SKKDS-NKFNLVDPVE--RNTVGVPSGGW--- 505
LAC11   456 --SHPFHLHGYNFFVV----------GTGVGNFD----PKKDP-AKFNLVDPPE--RNTVGVPTGGW--- 504
LAC12   464 --NHPIHLHGYDFYIV----------GEGFGNFN----PKKDT-SKFNLVDPPL--RNTVAVPVNGW--- 512
LAC13   468 --NHPMHLHGYEFYVV----------GTGVGNFN----PNTDT-SSFNLIDPPR--RNTIGTPPGGW--- 516
LAC14   468 --IHPIHLHGYNFYVV----------GSGFGNFD----RRKDP-LRYNLVDPPE--ETTVGVPRNGW--- 516
LAC15   459 -LDHPMHLHGFSFYVV----------GVGFGNYN---ISEEDPSSRYNLYDPPY--KNTMTVPRNGW--- 510
LAC16   422 --NHPFHLHGFNFFEV----------GRGLGNFN----PEKDP-KAFNLVDPVE--RNTVGVPAGGW--- 470
LAC17   476 --SHPLHLHGFNFFVV----------GQGFGNFD----PNKDP-RNFNLVDPIE--RNTVGVPSGGW--- 524
AO      480 SETHPWHLHGHDFWVL----------GYGEGKFN----ESEDP-KRYNRVDPIK--KNTVAVQPFGW--- 529
LPR1    462 --NHPLHIHLGLFKVVEQTAL----LAAGLEEFKECMTKQNDA-VKCQISKYARGKKTAVTAHERGWKNV 525
LPR2    464 --NHPLHIHLGLFKVLEQTAL----VKS--EEFIECMTKRNDA-VKCEISKYARGNKTAVTVHERGWKNV 525

ScFET3  467 ----------FVIRFK------------ADNPGVWFFHCHIEWHLLQGLGLVLVEDPFGIQDAHSQQLS- 514
LAC1    528 ----------AAIRIN------------ADNPGVWFIHCHLEQHTSWGLAMGFI-----VKDGPLPSQTL 571
LAC2    520 ----------VAIRFL------------ADNPGVWLMHCHIEIHLSWGLTMAWV-----VLDGDLPNQKL 563
LAC3    517 ----------VAIRFV------------ADNPGAWFMHCHIDSHLGWGLAMVFL-----VENGRGQLQSV 560
LAC4    505 ----------VVIRFR------------ADNPGVWFMHCHLEVHTTWGLKMAFL-----VENGKGPNQSI 548
LAC5    527 ----------AVIRFI------------ADNPGVWIMHCHLDAHISWGLAMAFL-----VENGNGVLQTI 570
LAC6    516 ----------AAIRFV------------ANNPGLWLLHCHFDIHQTWGMSTMFI-----VKNGKKVQESL 559
LAC7    514 ----------AVIRFT------------ANNPGAWIFHCHIDVHLPFGLGMIFV-----VKNGPTKSTTL 557
LAC8    513 ----------VVLRFI------------ANNPGLWLFHCHMDAHLPYGIMSAFI-----VQNGPTPETSL 556
LAC9    515 ----------VVLRFI------------ANNPGIWLFHCHMDAHLPLGIMMAFI-----VQNGPTRETSL 558
LAC10   505 ----------AAIRFR------------ADNPGVWFMHCHLEVHTTWGLKMAFL-----VENGKGPNQSI 548
LAC11   504 ----------AAIRFR------------ADNPGVWFMHCHLEVHTMWGLKMAFV-----VENGETPELSV 547
LAC12   512 ----------AVIRFV------------ADNPGVWLMHCHLDVHIKWGLAMAFL-----VDNGVGELETL 555
LAC13   516 ----------VAIRFV------------ANNPGAWLMHCHIDSHIFWGLAMVFL-----VENGEGHLQSV 559
LAC14   516 ----------TAVRFV------------ANNPGVWLLHCHIERHATWGMNTVFI-----VKDGPTKSSRM 559
LAC15   510 ----------IAIRFV------------ADNPGVWFMHCHLDRHQTWGMNVVFI-----VKNGREPNQQI 553
LAC16   470 ----------TAIRFI------------ADNPGVWFMHCHLELHTTWGLKMAFV-----VDNGHGPDQSL 513
LAC17   524 ----------AAIRFL------------ADNPGVWFMHCHLEVHTSWGLRMAWL-----VLDGDKPDQKL 567
AO      529 ----------TALRFR------------ADNPGVWSFHCHIESHFFMGMGIVF-------ESGIDKVSSL 570
LPR1    526 FKMMPGHVTRILVRFSYIHTNASYPFDPTQEPG-YVYHCHILDHEDNMMMRPLKV----II--------- 581
LPR2    526 FKMMPGHVTKILVRFSYIHSNESYSFDATQEPG-YVYHCHILDHEDNMMMRPFAM----VL--------- 581

ScFET3  514 ---ENHLEVCQSCSVATEGNAAANTLDLTDLTGENVQHAFIPTGFTKKGIIAMTFSCFAGILGIITIAIY 581
LAC1    572 LPPPHDLPQC------------------------------------------------------------ 581
LAC2    564 LPPPSDFPKC------------------------------------------------------------ 573
LAC3    561 QAPPLDLPRC------------------------------------------------------------ 570
LAC4    549 LPPPKDLPKC------------------------------------------------------------ 558
LAC5    571 EQPPHDLPVC------------------------------------------------------------ 580
LAC6    560 PHPPADLPKC------------------------------------------------------------ 569
LAC7    558 PPPPPDLPKC------------------------------------------------------------ 567
LAC8    557 PSPPSNLPQC------------------------------------TR---------------------- 568
LAC9    559 PSPPSNLPQC------------------------------------TR---------------------- 570
LAC10   549 RPPPSDLPKC------------------------------------------------------------ 558
LAC11   548 LPPPKDYPSC------------------------------------------------------------ 557
LAC12   556 EAPPHDLPIC------------------------------------------------------------ 565
LAC13   560 QSPPLDLPQC------------------------------------------------------------ 569
LAC14   560 VKPPPDLPSC------------------------------------------------------------ 569
LAC15   554 LPPPDDLPPCY----------------------------------------------------------- 564
LAC16   514 LPPPADLPKC------------------------------------------------------------ 523
LAC17   568 LPPPADLPKC------------------------------------------------------------ 577
AO      570 ---PSSIMGC---------------------------------GQTKR---------------------- 582
LPR1    581 ---------------------------------------------------------------------- 581
LPR2    581 ---------------------------------------------------------------------- 581
```

```
ScFET3  582 GMMDMEDATEKVIRDLHVDPEVLLNEVDENEERQVNEDRHSTEKHQFLTKAKRFF 636
LAC1    581 ------------------------------------------------------- 581
LAC2    573 ------------------------------------------------------- 573
LAC3    570 ------------------------------------------------------- 570
LAC4    558 ------------------------------------------------------- 558
LAC5    580 ------------------------------------------------------- 580
LAC6    569 ------------------------------------------------------- 569
LAC7    567 ------------------------------------------------------- 567
LAC8    568 ------------------DPTIY-------DSRTTNIDLS--------------Y 584
LAC9    570 ------------------DPTIY-------DSRTTNVDMS--------------Y 586
LAC10   558 ------------------------------------------------------- 558
LAC11   557 ------------------------------------------------------- 557
LAC12   565 ------------------------------------------------------- 565
LAC13   569 ------------------------------------------------------- 569
LAC14   569 ------------------------------------------------------- 569
LAC15   564 ------------------------------------------------------E 565
LAC16   523 ------------------------------------------------------- 523
LAC17   577 ------------------------------------------------------- 577
AO      582 ------------------------------------------------------- 582
LPR1    581 ------------------------------------------------------- 581
LPR2    581 ------------------------------------------------------- 581
```

Supplementary Figure 1. Amino acid sequence alignment of ScFET3 with Arabidopsis LMCO Oxidase family proteins and Ascorbate Oxidase. Conserved histidine residues involved in Cu binding are highlighted in green, and aspartic acid residues that putatively correspond to those known to be essential for ferroxidase activity of ScFET3 are highlighted in red (D319, D320) or in pink (E185, D283, D409) (Bonaccorsi di Patti et al., 2005; Quintanar et al., 2007). ScFET3, 855080. LAC1, At1g18140. LAC2, At2g29130. LAC3, At2g30210. LAC4, At2g38080. LAC5, At2g40370. LAC6, At2g46570. LAC7, At3g09220. LAC8, At5g01040. LAC9, At5g01050. LAC10, At5g01190. LAC11, At5g03260. LAC12, At5g05390. LAC13, At5g07130. LAC14, At5g09360. LAC15, At5g48100. LAC16, At5g58910. LAC17, At5g60020. AO, At4g39830. LPR1, At1g23010. LPR2, At1g71040.
