# Supplementary material for: Involvement of Arabidopsis Multi-Copper Oxidase-Encoding LACCASE12 in Root-to-Shoot Iron Partitioning: A Novel Example of Copper-Iron Crosstalk
Source: Front Plant Sci. 2021 Oct 11;12:688318. doi: 10.3389/fpls.2021.688318 (PMC8544784; doi:10.3389/fpls.2021.688318)
Supplement: Supplementary file 1 [file Data_Sheet_1.zip › Supplementary Figures 2-6.pptx]

## Slide 1
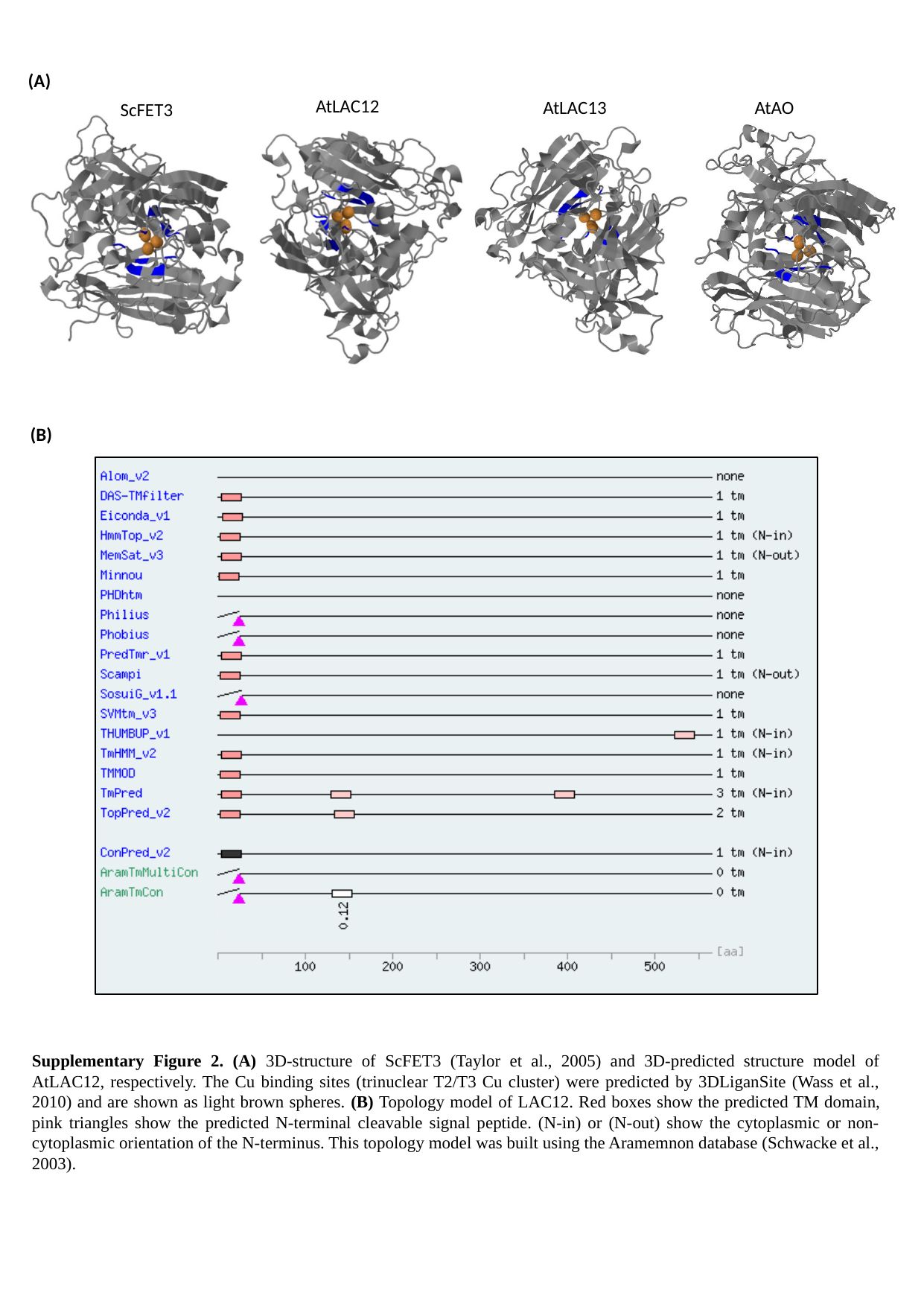

(A)
AtLAC12
AtLAC13
AtAO
ScFET3
(B)
Supplementary Figure 2. (A) 3D-structure of ScFET3 (Taylor et al., 2005) and 3D-predicted structure model of AtLAC12, respectively. The Cu binding sites (trinuclear T2/T3 Cu cluster) were predicted by 3DLiganSite (Wass et al., 2010) and are shown as light brown spheres. (B) Topology model of LAC12. Red boxes show the predicted TM domain, pink triangles show the predicted N-terminal cleavable signal peptide. (N-in) or (N-out) show the cytoplasmic or non-cytoplasmic orientation of the N-terminus. This topology model was built using the Aramemnon database (Schwacke et al., 2003).

## Slide 2
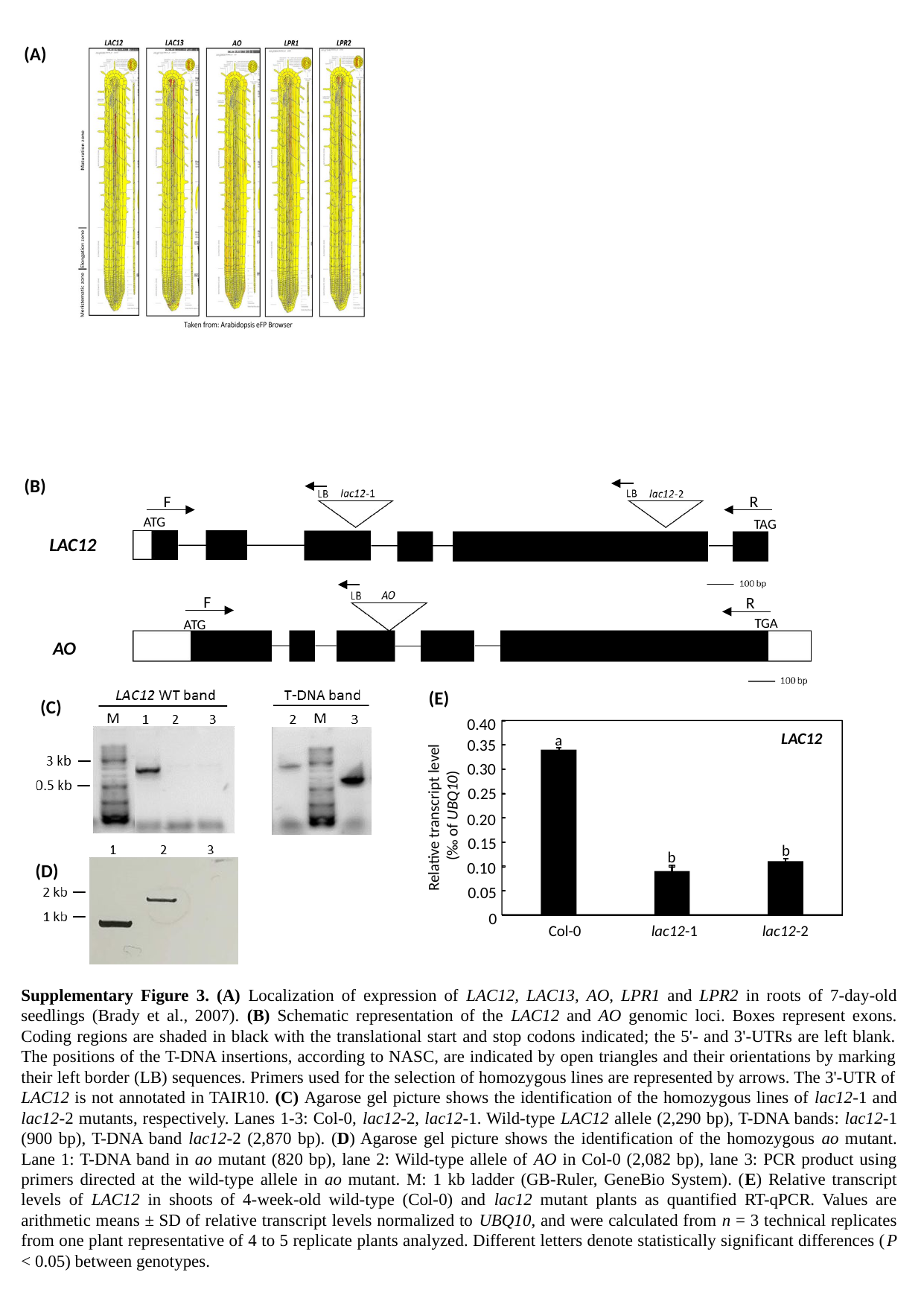

(A)
(B)
R
F
ATG
TAG
LAC12
F
R
TGA
ATG
AO
(E)
(C)
0.40
LAC12
a
0.35
0.30
0.25
Relative transcript level
 (‰ of UBQ10)
0.20
0.15
b
b
0.10
0.05
0
Col-0
lac12-1
lac12-2
(D)
Supplementary Figure 3. (A) Localization of expression of LAC12, LAC13, AO, LPR1 and LPR2 in roots of 7-day-old seedlings (Brady et al., 2007). (B) Schematic representation of the LAC12 and AO genomic loci. Boxes represent exons. Coding regions are shaded in black with the translational start and stop codons indicated; the 5'- and 3'-UTRs are left blank. The positions of the T-DNA insertions, according to NASC, are indicated by open triangles and their orientations by marking their left border (LB) sequences. Primers used for the selection of homozygous lines are represented by arrows. The 3'-UTR of LAC12 is not annotated in TAIR10. (C) Agarose gel picture shows the identification of the homozygous lines of lac12-1 and lac12-2 mutants, respectively. Lanes 1-3: Col-0, lac12-2, lac12-1. Wild-type LAC12 allele (2,290 bp), T-DNA bands: lac12-1 (900 bp), T-DNA band lac12-2 (2,870 bp). (D) Agarose gel picture shows the identification of the homozygous ao mutant. Lane 1: T-DNA band in ao mutant (820 bp), lane 2: Wild-type allele of AO in Col-0 (2,082 bp), lane 3: PCR product using primers directed at the wild-type allele in ao mutant. M: 1 kb ladder (GB-Ruler, GeneBio System). (E) Relative transcript levels of LAC12 in shoots of 4-week-old wild-type (Col-0) and lac12 mutant plants as quantified RT-qPCR. Values are arithmetic means ± SD of relative transcript levels normalized to UBQ10, and were calculated from n = 3 technical replicates from one plant representative of 4 to 5 replicate plants analyzed. Different letters denote statistically significant differences (P < 0.05) between genotypes.

## Slide 3
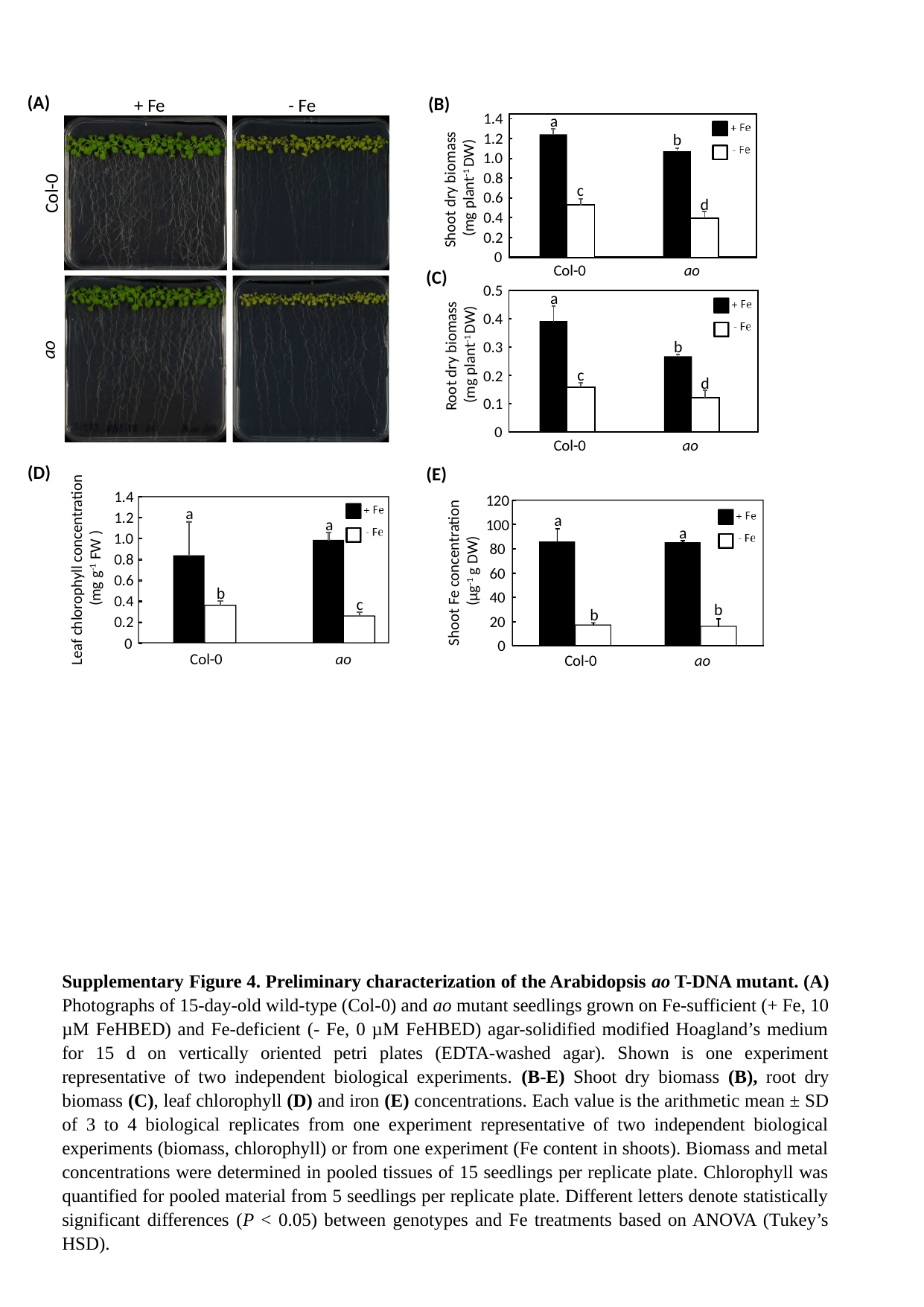

(A)
(B)
- Fe
+ Fe
a
1.4
b
1.2
1.0
Shoot dry biomass
(mg plant-1 DW)
0.8
c
0.6
d
0.4
0.2
0
Col-0
ao
Col-0
(C)
0.5
a
0.4
Root dry biomass
(mg plant-1 DW)
b
0.3
c
0.2
d
0.1
0
Col-0
ao
ao
(D)
(E)
1.4
a
1.2
a
1.0
Leaf chlorophyll concentration
(mg g-1 FW )
0.8
0.6
b
c
0.4
0.2
0
Col-0
ao
120
a
100
a
80
Shoot Fe concentration
 (µg-1 g DW)
60
40
b
b
20
0
Col-0
ao
Supplementary Figure 4. Preliminary characterization of the Arabidopsis ao T-DNA mutant. (A) Photographs of 15-day-old wild-type (Col-0) and ao mutant seedlings grown on Fe-sufficient (+ Fe, 10 µM FeHBED) and Fe-deficient (- Fe, 0 µM FeHBED) agar-solidified modified Hoagland’s medium for 15 d on vertically oriented petri plates (EDTA-washed agar). Shown is one experiment representative of two independent biological experiments. (B-E) Shoot dry biomass (B), root dry biomass (C), leaf chlorophyll (D) and iron (E) concentrations. Each value is the arithmetic mean ± SD of 3 to 4 biological replicates from one experiment representative of two independent biological experiments (biomass, chlorophyll) or from one experiment (Fe content in shoots). Biomass and metal concentrations were determined in pooled tissues of 15 seedlings per replicate plate. Chlorophyll was quantified for pooled material from 5 seedlings per replicate plate. Different letters denote statistically significant differences (P < 0.05) between genotypes and Fe treatments based on ANOVA (Tukey’s HSD).

## Slide 4
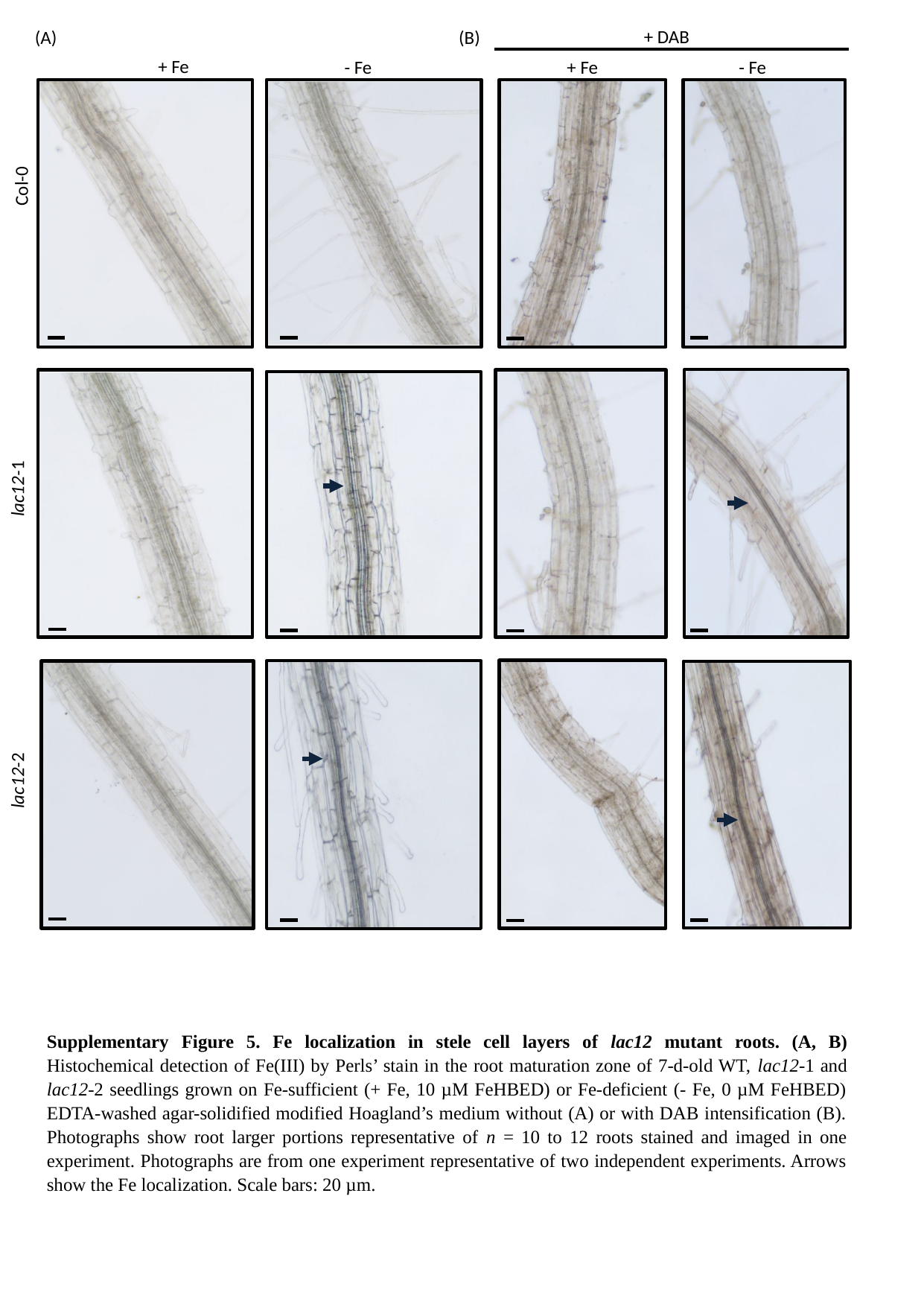

+ DAB
(A)
(B)
+ Fe
- Fe
+ Fe
- Fe
Col-0
lac12-1
lac12-2
Supplementary Figure 5. Fe localization in stele cell layers of lac12 mutant roots. (A, B) Histochemical detection of Fe(III) by Perls’ stain in the root maturation zone of 7-d-old WT, lac12-1 and lac12-2 seedlings grown on Fe-sufficient (+ Fe, 10 µM FeHBED) or Fe-deficient (- Fe, 0 µM FeHBED) EDTA-washed agar-solidified modified Hoagland’s medium without (A) or with DAB intensification (B). Photographs show root larger portions representative of n = 10 to 12 roots stained and imaged in one experiment. Photographs are from one experiment representative of two independent experiments. Arrows show the Fe localization. Scale bars: 20 µm.

## Slide 5
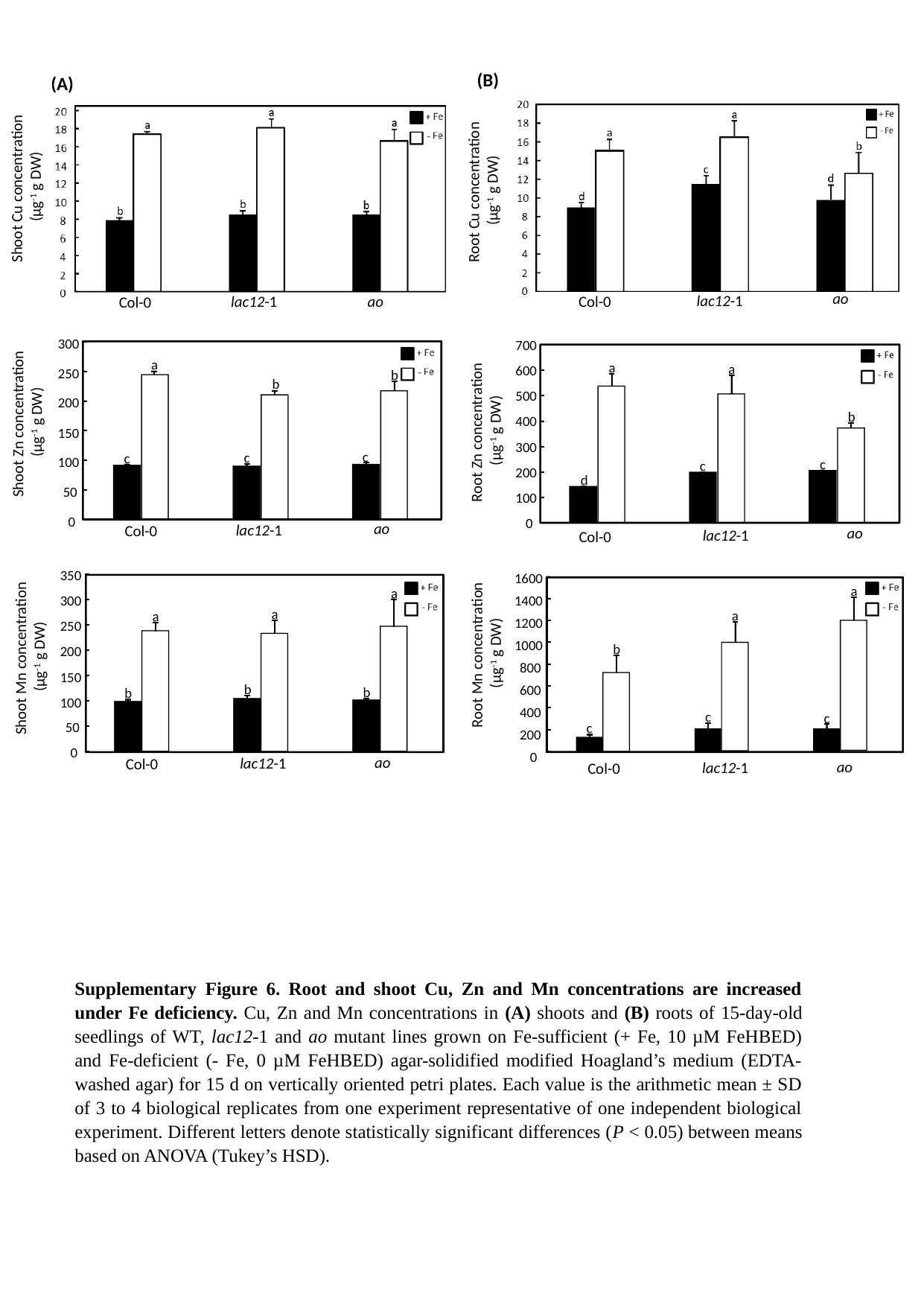

(B)
(A)
Shoot Cu concentration
 (µg-1 g DW)
Root Cu concentration
 (µg-1 g DW)
ao
lac12-1
lac12-1
Col-0
ao
Col-0
300
700
a
a
a
b
600
250
b
500
200
Shoot Zn concentration
 (µg-1 g DW)
b
Root Zn concentration
 (µg-1 g DW)
400
150
300
c
c
c
c
c
100
200
d
50
100
0
0
ao
lac12-1
Col-0
ao
lac12-1
Col-0
350
1600
a
a
1400
300
a
a
a
1200
250
Root Mn concentration
 (µg-1 g DW)
Shoot Mn concentration
 (µg-1 g DW)
b
1000
200
800
150
b
b
b
600
100
c
400
c
c
50
200
0
0
ao
lac12-1
Col-0
ao
lac12-1
Col-0
Supplementary Figure 6. Root and shoot Cu, Zn and Mn concentrations are increased under Fe deficiency. Cu, Zn and Mn concentrations in (A) shoots and (B) roots of 15-day-old seedlings of WT, lac12-1 and ao mutant lines grown on Fe-sufficient (+ Fe, 10 µM FeHBED) and Fe-deficient (- Fe, 0 µM FeHBED) agar-solidified modified Hoagland’s medium (EDTA-washed agar) for 15 d on vertically oriented petri plates. Each value is the arithmetic mean ± SD of 3 to 4 biological replicates from one experiment representative of one independent biological experiment. Different letters denote statistically significant differences (P < 0.05) between means based on ANOVA (Tukey’s HSD).
